# In Vitro Antiglycation Potential of Erva-Baleeira (*Varronia curassavica* Jacq.)

**DOI:** 10.3390/antiox12020522

**Published:** 2023-02-19

**Authors:** Winner Duque Rodrigues, Felipe Nunes Cardoso, Amanda Martins Baviera, André Gonzaga dos Santos

**Affiliations:** 1 Department of Drugs and Medicines, School of Pharmaceutical Sciences, São Paulo State University, Araraquara 14800-903, Brazil; 2 Department of Clinical Analysis, School of Pharmaceutical Sciences, São Paulo State University, Araraquara 14800-903, Brazil

**Keywords:** *Cordia verbenacea*, antiglycation activity, oxidative stress, cordialin A, brickellin

## Abstract

**Simple Summary:**

Natural products are interesting therapeutic options for the complementary treatment of chronic diseases, such as diabetes mellitus and its complications. In this study, the extract of Erva-Baleeira (*Varronia curassavica* Jacq.) and two substances isolated from its leaves were submitted to assays that simulated possible aggressions caused to proteins due to high amounts of glucose in the blood, typical of diabetes. Our findings suggest that all samples evaluated decreased the generation of reactive species and their damages to proteins, information that is useful to the understanding of the therapeutic properties of this plant species.

**Abstract:**

Background: *Varronia curassavica* Jacq. (Boraginaceae) is traditionally used in the treatment of inflammatory processes. The ethanolic extract of its leaves (EEVc) showed anti-inflammatory properties and low toxicity. Medicinal plants have aroused interest for their antiglycation activities. The formation and accumulation of advanced glycation end products (AGEs) are associated with several chronic diseases. The objective of this study was to evaluate the antiglycation potential of EEVc and two isolated compounds. Methods: The compounds brickellin and cordialin A were obtained by chromatographic methods and identified by spectrometric techniques. Analysis of fluorescent AGEs, biomarkers of amino acid residue oxidation, protein carbonyl groups and crosslink formation were performed in samples obtained from an in vitro model system of protein glycation with methylglyoxal. Results: EEVc, brickellin and cordialin A significantly reduced the in vitro formation of AGEs, and reduced the damage caused by oxidative damage to the protein. Conclusions: According to the results, EEVc, brickellin and cordialin A are potential candidates against AGEs formation, which opens the way to expand the therapeutic arsenal for many pathologies resulting from glycoxidative stress.

## 1. Introduction

The use of medicinal plants and natural products as a therapeutic resource is already well-known and contributes significantly to the development of drugs and medicines [1]. Among the native plants in traditional use in Brazil, the leaves or aerial parts of erva-baleeira (*Varronia curassavica* Jacq. sin. *Cordia verbenacea* DC., Boraginaceae family), may be highlighted due to their traditional use in the treatment of infections, gastric ulcers, pain, inflammation and rheumatism, as infusions and ethanolic or hydroethanolic extracts [2]. This species is available in popular fairs and is employed in phytotherapy programs of the Brazilian Public Health System, such as the “Farmácias Vivas” [3,4]. In addition, it is included in the official compendia of the country that aim to guide and stimulate studies on its use [5,6].

Different therapeutic activities have already been attributed to the compounds of the ethanolic and hydroethanolic extracts from its leaves, such as antioxidant, anti-inflammatory and antiedematous activities [7,8], in addition to a potent activity in the prevention and reduction in ethanol-induced gastric ulcers [9,10], and the extracts have been safe in terms of toxicity and teratogenicity in different animal models [11]. The essential oil of the leaves has anti-inflammatory activity and is the active ingredient of the topical herbal medicine Acheflan^®^ [12].

Medicinal plants are therapeutic alternatives for treating chronic inflammatory diseases, such as rheumatoid arthritis, osteoarthritis, atherosclerosis, diabetes mellitus and neurodegeneration [13,14]. The inflammatory process is complex and modulated in many ways and plays essential physiological roles in the body [15]. However, when exacerbated, its damage leads to harm, modifying the function and integrity of macromolecules, increasing the levels of reactive oxygen species and causing oxidative stress, which is associated with the physiopathology of several chronic inflammatory diseases [16].

Within this context, the present study focused on evaluating the effect of *V. curassavica* extract and isolated metabolites on the formation of advanced glycation end-products (AGEs). AGEs are products generated from the interaction of reducing sugars with amino groups of proteins, being formed mainly by the endogenous route, occurring under physiological conditions in all tissues and body fluids [17]. Their accumulation in the human body is favored in conditions of chronic hyperglycemia, aging, dietary patterns, impaired AGE detoxification mechanisms, and unhealthy lifestyles such as sedentary lifestyles, smoking and chronic stress. The presence and accumulation of AGEs in the body contribute to the triggering and aggravation of several chronic diseases and metabolic syndromes [18]. In addition, AGEs negatively interfere with the chemical and functional properties of several proteins through the formation of intra- and inter-protein crosslinks, and interactions with cellular receptors, favoring the generation of free radicals [19,20]. AGE interaction with its specific receptor, named RAGE (Receptor for Advanced Glycation End-products) causes an increased expression of pro-inflammatory cytokines and exacerbation in the production of reactive oxygen species in cells, which results in increased inflammation and oxidative stress [21,22,23].

Several mechanisms have been exploited to intervene in the formation, accumulation and oxidative damage caused by AGEs in the body [16]. To date, there are still no drugs on the market that act directly on AGEs. It is known that some compounds have antiglycation activity, but further studies are still needed. One example is aminoguanidine, which has exceptional antiglycation activity, but clinical studies have found the presence of relevant adverse effects, leading its therapeutic use to be discontinued [24].

Recent reviews demonstrate that medicinal plants and natural products have been relevant in the prospection of new therapeutic agents with antiglycation activity [15,25]. Thus, using an in vitro model system of protein glycation, the present study aimed to investigate the antiglycation effects of *V. curassavica* leaf extract and two isolated compounds, brickellin and cordialin A.

## 2. Materials and Methods

### 2.1. Plant Material and Extract

The harvesting of the leaves, the processing and the extraction, were conducted by Dr. Juhan A. Scardelato Pereira. The leaves of *Varronia curassavica* Jacq. were collected at the experimental field of Pluridisciplinary Center for Chemical, Biological and Agricultural Research (CPQBA) of the University of Campinas–Unicamp, Betel District, Paulínia city, São Paulo state, Brazil (22°47′15.91″ S 47°06′42.87″ W) on 10/31/2013 at 12:00 am (27 °C, relative humidity of 65%, cloudy weather). A voucher specimen was deposited at the Herbarium São José do Rio Preto (IBILCE-Unesp, São José do Rio Preto, São Paulo state, Brazil) under the number 31235. The leaves were dried in an oven (40 °C) with air circulation for 3 days and powdered in a knife mill. The extraction was performed with ethanol by remaceration in 3 steps at 35 °C under occasional stirring. The extraction time in the first step was 24 h, and in the last two, 48 h each. The total plant drug: solvent ratio was 1:15 (1 kg/15 L).

### 2.2. Experimental

The solid phase extraction (SPE) of the extract was performed with silica gel (60–200 μm, Sigma-Aldrich, St. Louis, MI, USA) as the stationary phase. Thin layer chromatography (TLC) was performed on silica gel chromatoplates (0.20 µm, 60G, F254, Macherey-Nagel, Germany). The analytical grade solvents employed in SPE and TLC were hexane (Synth, São Paulo, Brazil), chloroform, ethyl acetate and methanol (Qhemis, São Paulo, Brazil). The TLC spray reagent was composed by sulfuric acid and ethanol (Synth, São Paulo, Brazil). For the high-performance liquid chromatography (HPLC) sample pretreatment by SPE, a C18 cartridge (45 μm; 500 mg; 6 mL, SampliQ, Agilent Technologies, USA) was employed. For the HPLC analyses and sample pretreatment, the samples were prepared with HPLC grade methanol (J.T. Baker, CA, USA) and ultrapure water (MilliQ, Merck, Germany) and filtered through PVDF filter membranes (0.22 μm, Sigma-Aldrich, St. Louis, MI, USA). The analyses were performed in HPLC-UV (Thermo Scientific Ultimate 3000, Waltham, MA, USA) coupled to a C18 column (250 × 4.6 mm; 5 μm, Thermo Sientific, Waltham, MA, USA). Compounds isolation was performed on HPLC-UV (Perkin Elmer Flexar, Waltham, MA, USA) coupled to a C18 semipreparative column (250 × 21.20 mm; 7 μm, Eclipse XDB, Agilent Technologies, Santa Clara, CA, USA). The Fourier Transform Infrared (FTIR) spectra were obtained using a spectrometer (VERTEX 70, Bruker, Billerica, MA, USA) in Attenuated Total Reflectance (ATR) mode from 400 to 4000 cm^−1^ at 4 cm^−1^ resolution and 32 scans. The ultraviolet (UV) spectra were obtained (Synergy TM H1, BioTek Instruments Inc., Winooski, VE, USA) at wavelength range of 210–450 nm. The identification of the purified compounds was based on the spectrometric data from ^1^H (400 MHz) and ^13^C (100 MHz) one-dimensional nuclear magnetic resonance spectrometry (model DRX400, 9.4 T–Ultra Shield, Bruker, USA) with deuterochloroform (Sigma Aldrich, St. Louis, MI, USA) as solvent and TMS as internal reference. Mass spectrometry (HPLC-MS Acquity QDa, Waters, Milford, MA, USA) were performed in the full-scan analysis mode and monitored by the masses of cationized molecules with sodium [M + Na]^+^ of *m/z* 400 to 600.

#### Compounds Isolation and Purification

The fractionation of the dry extract (8 g) was performed by SPE in a glass column (12 × 10 cm) containing silica gel. The elution was developed under reduced pressure as follows (eluent volume of 900 mL and fractions volume of 300 mL): (a) hexane: ethyl acetate 8:2 (*v*/*v*)–fractions: F1.1; F1.2; F1.3; (b) 6:4 (*v*/*v*)–F2.1; F2.2; F2.3; (c) ethyl acetate–F3.1; F3.2; F3.3; (d) ethyl acetate: methanol 9:1 (*v*/*v*)–F4.1; F4.2; F4.3; (d) methanol–F5.1; F5.2; F5.3.

The SPE separation was evaluated by TLC and HPLC-UV. TLC: The fractions and the EEVc solutions (5 mg/mL, ethyl acetate) were applied (40 µL) in silica gel chromatoplates using as mobile phases: (a) chloroform: ethyl acetate: methanol 5.5:3.5:1 (*v*/*v*); (b) chloroform: ethyl acetate 6:4 (*v*/*v*). The spray reagent was 10% sulfuric acid in ethanol (110 °C, 10 min). HPLC-UV: The fractions and the EEVc (10.0 mg sample in 1.0 mL of methanol: water 95:05 *v*/*v*) were submitted to SPE in a C18 cartridge and eluted with 4.0 mL of methanol: water 95:05 (*v*/*v*). The eluate was dried, solubilized in methanol (1.0 mg/mL), and filtered through a PVDF membrane. The samples (20 μL) were analyzed on HPLC-UV with a C18 column under a linear gradient of 5–100% methanol in 30 min plus methanol in 5 min; flow rate of 1.0 mL/min; detector wavelength at 254 nm.

F4.1 and F4.3 were selected for semipreparative HPLC separation. The fractions were submitted to a pretreatment similar to the analytical pretreatment, and aliquots of 1.0 mL of their solutions (50 mg/mL) were injected onto HPLC. The semipreparative separation was performed on HPLC-UV coupled to a C18 semipreparative column with methanol: water 75:25 isocratically for 35 min; flow rate of 8.0 mL/min; detector wavelength at 254 nm. The purity (area normalization method) of the isolated compounds was determined by HPLC-UV using the same equipment, coupled to C18 analytical column with methanol: water 75:25 isocratically for 35 min; flow rate of 0.4 mL/min; detector wavelength at 254 nm.

### 2.3. In Vitro Model System of Protein Glycation

The in vitro model system of protein glycation was in accordance with dos Santos et al. [26], with modifications. Bovine serum albumin (BSA: 10 mg/mL) (Sigma-Aldrich, St. Louis, MI, USA) was incubated in the presence of methylglyoxal (MGO: 5 mM), in phosphate buffer (0.1 M, pH 7.4) (Sigma-Aldrich, USA) containing 0.02% sodium azide (Merck, Germany), at 37 °C for 8 days. The incubations were conducted in the absence or in the presence of different concentrations of EEVc (62.5; 125; and 250 µg/mL) or brickellin (0.031; 0.062; and 0.125 mM) or cordialin A (0.025; 0.051; and 0.102 mM). Aminoguanidine (AG: 1 mM) (Sigma-Aldrich, St. Louis, MI, USA) was used as a prototype therapeutic agent with anti-AGE activity [27].

In the phosphate buffer, present in all tubes, dimethyl sulfoxide (DMSO: 5% *v*/*v*) (Sigma-Aldrich, St. Louis, MI, USA) was added, as it acted as a cosolvent in the solubilization of EEVc and its isolated compounds. The aliquots were collected on days 0, 1, 2, 4 and 8 to perform the determinations described below.

#### 2.3.1. Determination of Fluorescent AGE Formation

The monitoring of the fluorescent AGE generation was performed in a spectrofluorometer (Synergy TM H1, BioTek Instruments Inc., Winooski, VE, USA), at excitation and emission wavelengths of 355 and 430 nm, respectively [28]. The antiglycation potential of EEVc and the isolated compounds was evaluated by comparing the AGE-related fluorescence generated in the incubation of BSA + MGO against the AGE-related fluorescence generated in the incubation of BSA + MGO + EEVc or BSA + MGO + isolated compounds at different concentrations.

The fluorescence values relative to the AGEs were obtained after the arithmetic subtraction of the fluorescence of the incubations of the EEVc or isolated compounds with buffer from those of the EEVc or isolated compounds incubated with BSA or BSA + MGO. The results were expressed in terms of arbitrary units (A. U.) of fluorescence. The fluorescence intensities were measured using a microplate multimode reader (Synergy TM H1, BioTek Instruments Inc., USA).

#### 2.3.2. Determination of the Formation of Markers Related to Amino Acid Oxidation

Markers related to amino acid oxidation were monitored spectrofluorometrically (Synergy TM H1, BioTek Instruments Inc., Winooski, VE, USA), at the respective excitation/emission wavelengths: dityrosine (330/415 nm) as a marker of tyrosine residue oxidation; kynurenine (365/480 nm) and *N′*-formylkynurenine (325/434 nm) as markers of tryptophan residue oxidation [27].

The fluorescence values of dityrosine, kynurenine and *N′*-formylkynurenine were obtained after the arithmetic subtraction of the fluorescence of incubations of the EEVc or isolated compounds with buffer from those of the EEVc or isolated compounds incubated with BSA or BSA + MGO. The results were expressed in terms of arbitrary units (A. U.) of fluorescence.

#### 2.3.3. Quantification of Carbonylated Proteins

Carbonyl groups in proteins (PCO) are used as biomarkers of oxidative damage. The PCO levels were evaluated in reaction with 2,4-dinitrophenylhydrazine (DNPH), generating dinitrophenylhydrazone, monitored at 370 nm. The PCO levels were estimated using the molar extinction coefficient of the hydrazone (22,000 M^−1^·cm^−1^). Results were expressed in terms of μmol/L [29,30].

#### 2.3.4. Modification of BSA via Crosslink Formation

The formation of crosslinks in the proteins (protein crosslinking) was analyzed via polyacrylamide gel electrophoresis under denaturing conditions (SDS-PAGE) using incubation samples from day 0 and day 8 of the experiment. Briefly, 3 μL aliquots of the incubations were added to 9 μL of buffer containing 62.5 mM Tris-HCl (pH 6.8) (Sigma-Aldrich, USA), 10% glycerol, 2% sodium dodecyl sulfate (Sigma-Aldrich, USA), 100 mM dithiothreitol and 0.1% bromophenol blue. An aliquot (1.5 μL) of the resulting solution (which contain 3.75 μg of protein) was subjected to electrophoretic separation performed in SDS-PAGE (12%) for 1 h and 30 min at 120 V, in electrophoresis buffer [25 mM Tris-HCl (pH 8.3), 192 mM glycerol and 0.1% SDS] [26,31].

The gel was stained with Coomassie blue solution for 30 min and then underwent 3 washes with bleaching solution (10% methanol, 10% acetic acid), followed by staying 48 h in bleaching solution, in order to ensure a better visualization of the bands in the gel [26,31]. Densitometric calculations were performed using the Image J (v.1.53k) program on the bands generated in the crosslinking region in order to better measure the differences between the samples.

#### 2.3.5. Statistical Analysis

The results were expressed as means ± standard error of the mean (SEM) and were analyzed using One Way Analysis of Variance (ANOVA) followed by the analysis of difference by the Newman-Keuls test. The software used was GraphPad Prism 9. The level of statistical significance considered was *p* < 0.05.

## 3. Results and Discussion

### 3.1. Identification of Isolated Compounds

The compounds used in this work were isolated and purified through SPE followed by preparative HPLC-UV. The TLC and HPLC-UV analyses (Figure S1 Supplementary Material) of the SPE fractions showed a simple chromatographic profile for F4.1–3 and F5.1–3, and the purification of the substances directly by preparative HPLC-UV was feasible. The compounds were identified by one-dimensional nuclear magnetic resonance spectrometry (^1^H and ^13^C NMR), mass spectrometry (MS), infrared (IR) and ultraviolet (UV) absorption spectrophotometry. The spectrometry data were compared to the data in the literature [32,33,34,35,36].

Compound (**A**) was obtained as a crystalline, yellowish, circularly shaped powder with maximum UV absorptions (λ_max_) at 255 and 348 nm. The absorptions in the IR region were characteristic of angular and axial deformations corresponding to the functional groups: the range of 3200 cm^−1^ represents the stretching of O-H; the two intense bands between 1702 and 1200 cm^−1^ represent C–O and C–OH stretching. The range of 3000–2800 cm^−1^ is the location of bands connected with the asymmetric and symmetric stretching modes of C–H: ν_as_ (CH_3_), ν_s_ (CH_3_), ν_as_ (CH_2_) and ν_s_ (CH_2_), arising from the methyl and methylene groups, and a tetrasubstituted alkene conjugated with aromatic ring and carbonyl in 1604 cm^−1^. In the range of 900–690 cm^−1^, bands can be seen related to the stretching and bending vibrations of C–H from the aromatic rings [37]. The mass spectra obtained by HPLC-MS in the positive ion mode (ESI^+^) presented a peak at *m/z* 427.0 [M + Na]^+^ and in the negative ion mode (ESI^−^), at *m/z* 403.2 [M − H]^−^ (Figure S2 Supplementary Material).

The ^13^C NMR spectrum showed 20 signals, being consistent with the structure of the fundamental core of pentamethoxylated flavonoid compounds. The signals observed in the ^13^C NMR spectra (Table 1) with a chemical shift (*δ*) of 62.1 *q*, 60.8 *q*, 56.0 *q*, 56.4 *q* and 56.8 *q* and ^1^H NMR at *δ* 3.89 *s*, 3.93 *s*, 3.97 *s*, 3.94 *s* and 3.93 *s* were attributed to the presence of five methoxyl groups. The signals with *δ* values in the ^13^C NMR spectrum typical of oxygenated aromatic carbons at *δ* 152.8 *s* and 151.3 *s* were assigned to the carbons attached to the phenolic hydroxyls; the signals of the two hydrogens of the hydroxyls were observed at *δ* 12.38 *s* and 7.90 *s*. The positions of the substituent groups (five methoxyl and two hydroxyl groups) on the aromatic rings (A and B) were assigned based on the multiplicity of the remaining hydrogens on the aromatic rings (all singlets), in comparison with the ^13^C NMR data of polymethoxylated flavones described in the literature [32,34,35]. The signals at *δ* 103.0 *d* and 111.2 *d* observed in the ^13^C NMR spectrum and at *δ* 6.64 *s* (^1^H) and 7.11 *s* (^1^H) in the ^1^H NMR spectrum were assigned to the 3′ and 6′ positions of the B ring. The signals at *δ* 6.52 *s* in the ^1^H NMR spectrum and at *δ* 90.67 *d* in the ^13^C NMR spectrum correspond to the unsubstituted carbon of ring A (C8) and its corresponding hydrogen (H8). The signal with a chemical shift value of *δ* 177.4 *s* in the ^13^C NMR spectrum showed the presence of the carbonyl, and the signals at *δ* 136.6 *s* (C3) and 155.9 *s* (C2) refer to the double bond in the C ring.

**Figure 1:**
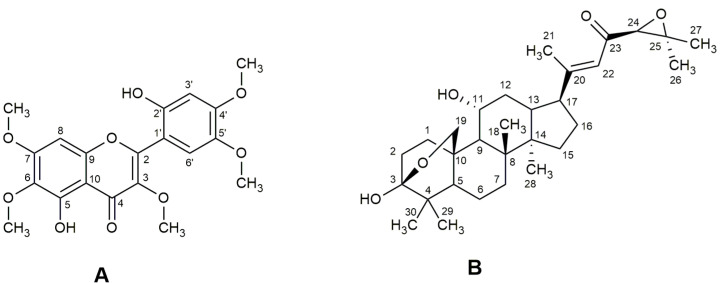
Chemical structures of brickellin (**A**) and cordialin A (**B**).

These analyses were conducted for the identification of 4′,5-dihydroxy-2′,3,5′,6,7-pentamethoxyflavone or 2′,5-dihydroxy-4′,3,5′,6,7-pentamethoxyflavone (brickellin). The ^13^C NMR data were similar to brickellin (Figure 1A), isolated for the first time from *Brickellia veronicaefolia* (HBK) Gray [33]. On the other hand, the differences in the *δ* values for 4′,5-dihydroxy-2′,3,5′,6,7-pentamethoxyflavone were observed for C3, C4, C9, C10, C1′, C3′, C4′ and C5′ [34]. The ^1^H NMR data for H3′ and H6′ for the isolated flavone are also closer to the data for brickellin [34,35] than for 4′,5-dihydroxy-2′,3,5′,6,7-pentamethoxyflavone [34]. Velde et al. [36] isolated two pentamethoxyflavones identified from *V. curassavica* leaves as artemetin and 5,6′-dihydroxy-3,3′,4′,6,7-pentamethoxyflavone; the NMR data for the last flavone was not shown in this work, thus it is not possible to confirm if it is also brickellin. Table 1 shows the spectrometric data obtained for brickellin at 400 MHz for ^1^H and 100 MHz for ^13^C. The ^1^H and ^13^C NMR spectra of brickellin are presented in the supplementary material (Figures S3 and S4 Supplementary Material).

Compound (**B**) was isolated as a white and amorphous powder. Its UV spectrum showed a band with λ_max_ at 255 nm. The absorptions in the IR region were characteristic of angular and axial strains corresponding to the functional groups: hydroxyl (3452 cm^−1^); ketone (1704 cm^−1^); ether (1060 cm^−1^) [32,37], and with a trisubstituted double bond (1602 and 805 cm^−1^) characteristic of cordialin A (Figure 1B). The mass spectrum (HPLC-MS) in the positive ion mode (ESI^+^) presented a peak at *m/z* 509.0 [M + Na]^+^, consistent with a molecular mass of 486.0 Da of cordialin A (Figure S5 Supplementary Material).

The ^1^H and ^13^C NMR spectra data were coherent with the damarane-type triterpene core found by Velde et al. [36] and Pereira [32] (Table 2) and allowed us to identify cordialin A (Figure 1B). The signal with *δ* value in the ^13^C NMR spectrum at 98.3 *s* (C3) was assigned to the hemiacetal carbon in the A ring of the damarane-type core. The signals in the ^13^C NMR spectrum at *δ* 17.1 *q* (C21), 24.8 *q*, 18.6 *q*, 26.3 *q*, 18.8 *q* and 16.7 *q* (C26-C30) and in the ^1^H NMR spectrum at *δ* 1.89 *m*, 2.12 *m*, 1.43 *s*, 1.27 *d*, 1.04 *s*, 1.01 *s*, 0.91 *s* correspond to the seven methyl groups of cordialin A. The *δ* value in the ^13^C NMR spectrum of 195.6 *s* was assigned to the ketone group at C23. The eter group was assigned to the carbons C3 and C19, which showed *δ* values of 98.3 *s* and 67.8 *t* in the ^13^C NMR spectrum, respectively. The presence of the trisubstituted double bond at C20 and C22 was confirmed by the signals in the ^13^C NMR with *δ* values of 164.2 *s* and 120.3 *d*, respectively. The signals of the 24,25-epoxide group were observed in the ^13^C NMR spectrum at *δ* 66.3 *d* and 61.0 *s*, respectively. The relative configuration was proposed based on the comparison of the NMR data with the literature data and the analysis of the coupling constants observed in the ^1^H NMR spectrum. The ^1^H and ^13^C NMR spectra of cordialin A are presented in the supplementary material (Figures S6–S8 Supplementary Material).

### 3.2. Effects of EEVc, Brickellin and Cordialin A on Glycoxidation Changes in an In Vitro Model System of Protein Glycation

#### 3.2.1. Formation of Fluorescent AGEs and Markers of Oxidation of Amino Acid Residues

The deleterious effects caused by the exacerbated generation of AGEs in the body mainly affect long-lived proteins (hemoglobin, collagen, elastin and others), as well as short-lived proteins, such as plasma albumin. Albumin constitutes about 50% of the proteins present in the plasma of healthy individuals and is involved in several physiological processes, mainly in the transport of compounds [38]. Thus, in vitro model systems of protein glycation have been used to investigate the deleterious consequences of protein glycation, as well as to study the antiglycation activity of compounds or preparations. In practical terms, bovine serum albumin (BSA) has been a viable choice as it shows homology with human serum albumin (approximately 76%) [31,39].

**Figure 2:**
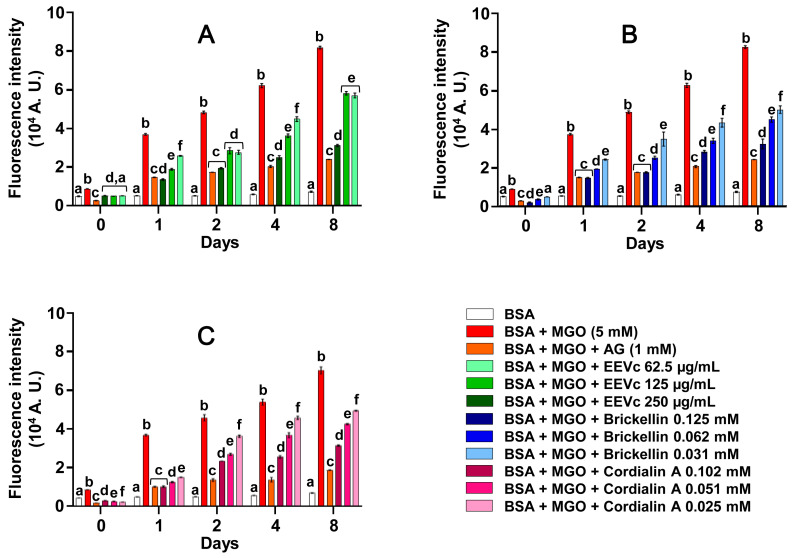
Effects of different concentrations of EEVc (**A**), brickellin (**B**) and cordialin A (**C**) on the formation of fluorescent AGEs in an in vitro model system of protein glycation with BSA + MGO. BSA: bovine serum albumin; MGO: methylglyoxal; AG: aminoguanidine; EEVc: ethanolic extract of *V. curassavica*. For all variables with the same letter, the difference between the means is not statistically significant.

The fluorescence intensities relative to AGE formation had progressive increases in incubations of BSA + MGO when compared to the corresponding values of BSA alone. Furthermore, BSA + MGO + AG had low levels of fluorescent AGEs, showing the responsiveness of this in vitro model system of protein glycation to interventions that are able to inhibit the protein damage due to glycation (Figure 2). The incubations of BSA + EEVc, brickellin and cordialin A (without MGO) did not emit significant fluorescence relative to the AGEs, showing fluorescence values comparable to those found in the incubations of BSA alone (Figure S9 Supplementary Material).

The absorption spectra graphs (Figures S10–S13) show data relative to the incubation samples of the controls, EEVc, brickellin and cordialin A. The EEVc and cordialin A in buffer without BSA had similar absorption values of BSA + MGO on days 0 and 1, which may be associated with the ability of cordialin A and/or compound(s) in EEVc to absorb light between 300 and 400 nm, and/or some kind of interaction with the buffer or the co-solvent, but on the other days, the absorption of BSA + MGO was more expressive, surpassing any tested sample and concentration.

When incubating the samples with the BSA + MGO, all tested samples decreased the fluorescence relative to the AGEs. In Figure 2A, it can be observed that EEVc, at all tested concentrations, inhibited AGE formation until the last day of the experiment. The highest concentration of EEVc (250 µg/mL) was the most efficient in protecting the BSA against glycation; on days 1 and 2 there was no significant difference between EEVc 250 µg/mL and AG (1 mM) in relation to the anti-AGE effect. Both compounds, brickellin (Figure 2B) and cordialin A (Figure 2C), significantly decreased the AGE formation during all periods of the study and at all tested concentrations. At the highest concentrations, these compounds showed a potential to inhibit AGE formation comparable to the AG for 1 and 2 days (for brickellin) and 1 day (for cordialin A). Furthermore, the anti-AGE activities of brickellin and cordialin A were found to have a concentration-dependent response. In the literature, it is described that medicinal plants and polyphenols have antiglycation activity and act on various targets that culminate in AGE redution in the body [15,25,39]. Plants belonging to the same family as the *V. curassavica* species, Boraginaceae, have shown antiglycation activities, including *Cordia platythyrsa* Baker [40] and *Cordia sinensis* Lam. [41].

Chinchansure et al. [39] consider that plants rich in phenolic compounds are beneficial against injuries caused by glycoxidative stress as they have antioxidant and antiglycation activities, which would result in a synergism against AGE formation and accumulation, as well as in reducing oxidative stress. In *V. curassavica*, compounds such as rosmarinic acid, caffeic acid, gallic acid and chlorogenic acid were identified, as well as the isoflavones 7,4′-dihydroxy-5′-carboxymethoxy-isoflavone and 7,4′-dihydroxy-5′-methyl-isoflavone; polymethoxylated flavones such as artemetin and 5,6′-dihydroxy-3,6,7,3′,4′-pentamethoxyflavone; and the triterpenes cordialin A, *(Z)*-cordialin A and cordialin B, [32,36,42,43,44,45,46,47,48]. Roldão [9] performed in vitro assays with the ethanolic extract of *V. curassavica*, which inhibited, in a concentration-dependent manner, the lipid peroxidation induced in rat hepatocyte plasma membranes. The EC_50_ was 76.11 ± 3.76 µg/mL, and quercetin, used as a positive control, was 4.58 ± 0.52 µg/mL. While another ethanolic extract of *V. curassavica*, analyzed by Santi et al. [10], showed a EC_50_ of 316.7 ± 23.16 µg/mL in the assay with DPPH and quercetin as the control (EC_50_= 2.33 ± 0.14 µg/mL). The total phenolic compounds content was 79.48 ± 0.63 mg GAE.g^−1^ (expressed as mg gallic acid equivalents per gram of extract).

Rohn [49] observed that polyphenols can interact with various proteins, including BSA, through covalent or non-covalent interactions, and among the amino acid residues susceptible to interaction with phenols, it can be cited to include the nucleophilic chains of lysine and cysteine, which are often associated with protein glycation [50]. Among the polyphenols, the class that commonly appears in studies are flavonoids, which can be justified given their wide distribution in plants; they have many biological activities, such as antioxidant and anti-inflammatory [51,52]. In the present study, brickellin at the concentration of 0.125 mM inhibited the formation of AGEs up to 2 days after the beginning of the experiment, without a significant difference with AG (1 mM), and maintained the anti-AGE activity in the following days, along with the other concentrations that proceeded in a concentration-dependent manner.

Brickellin is a flavone with a hydroxyl present on the B ring (C2′); studies relating the structure-activity of flavonoids against AGE formation show that the presence of hydroxyl groups on the A and B rings increases the ability to inhibit AGE formation [53] and that, in general, flavones show greater anti-AGE activity than flavonols, flavanones and isoflavones [51].

Triterpenes can inhibit the interaction of reducing sugars with proteins and, consequently, the AGE formation, decreasing oxidative stress [54]. Some known examples in the literature are: the astragaloside-type triterpenes that inhibit carboxymethylysine formation [55], ursolic acid and erythrodiol that have antioxidant and anti-inflammatory activities, these are believed to modulate glycation and to decrease inflammation and oxidative stress [56,57,58,59]. In the present study, cordialin A inhibited the AGE formation in a concentration-dependent response. As mentioned earlier, *V. curassavica* leaves contain the triterpenes cordialin A, *(Z)*-cordialin A and cordialin B [32,36]. The content of cordialin A in EEVc was 4.89% (m/m) in our study.

According to the data described in the literature, the efficiency of triterpenes on inhibiting the formation of AGEs in the body may be related to the interaction with the macromolecules involved in the glycation process [54]. For example, there are triterpenes, such as oleanolic and ursolic acid, that act by modulating the activity and/or the expression of some enzymes, including aldose reductase and sorbitol dehydrogenase (whose levels are decreased), resulting in the reduction of AGE formation, as well as increasing glyoxalase I, leading to the detoxification of AGE precursors and, thus, reducing their accumulation in the circulation and tissues [59,60]. The aforementioned triterpenes are not damarane-type as cordialins, but they have some structural similarities.

Another way that triterpenes may contribute to AGE reduction is related to their ability to scavenge free radicals, so contributing with non-enzymatic antioxidant compounds such as reduced glutathione, ascorbic acid and α-tocopherol in the body’s defense [57]. In addition, it has been observed that the triterpenoids lupeol and lupeol linoleate increased the activity of antioxidant enzymes, such as superoxide dismutase, catalase, glutathione peroxidase and glutathione S-transferase [61].

**Figure 3:**
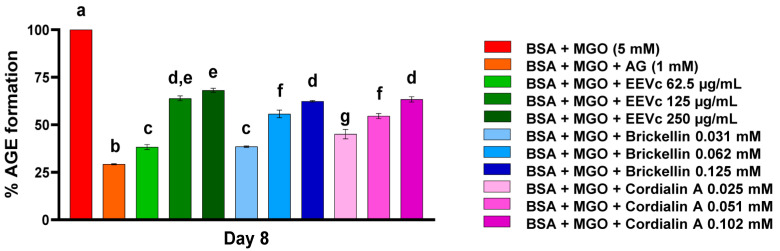
Percentage of AGE formation in an in vitro model system of protein glycation with BSA + MGO on day 8. BSA: bovine serum albumin; MGO: methylglyoxal; AG: aminoguanidine; EEVc: ethanolic extract of *V. curassavica*. For all variables with the same letter, the difference between the means is not statistically significant.

In general, EEVc and the isolated compounds, brickellin and cordialin A, at all tested concentrations, promoted the protection of BSA against the deleterious modifications caused by MGO, inhibiting AGE formation until the last day of analysis, day 8 (Figure 3). The AG used as prototype anti-AGE agent inhibited 70.7% of the AGE formation. AG is known to react with dicarbonyl compounds, such as MGO, to form triazines [24]. There was no statistically significant difference between EEVc 250 µg/mL and brickellin 0.125 mM when compared with the antiglycation activity of AG; both reduced the AGE formation by 61.7% and 60.8%, respectively. Cordialin A at the concentration of 0.102 mM was also able to inhibit AGE formation by more than half, equaling 54.9% (Figure 3).

**Figure 4:**
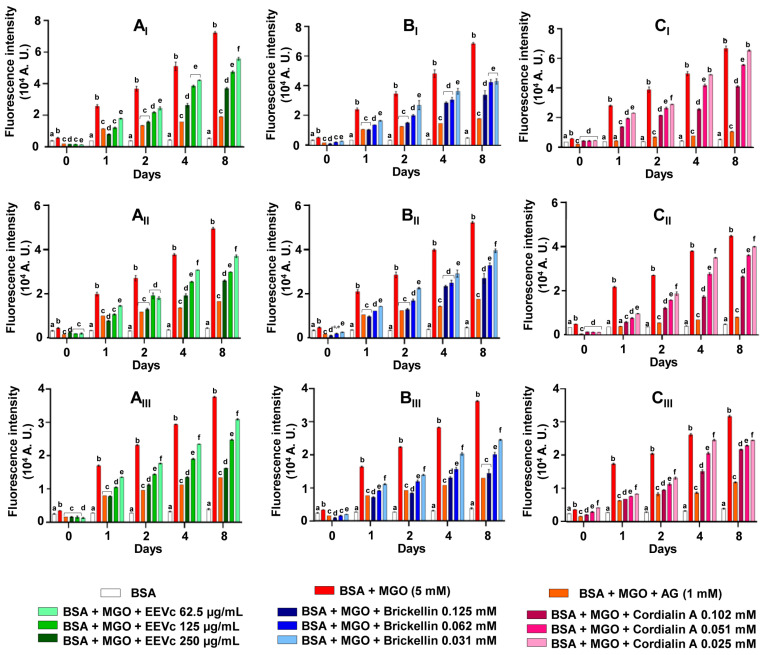
Effects of different concentrations of EEVc (**A**), brickellin (**B**) and cordialin A (**C**) on the formation of dithyrosine (I), *N′*-formylquinurenine (II) and quinurenine (III) in in vitro protein glycation model system with BSA + MGO. BSA: bovine serum albumin; MGO: methylglyoxal; AG: aminoguanidine; EEVc: ethanolic extract of *Varronia curassavica*. For all variables with the same letter, the difference between the means is not statistically significant.

Protein glycation is often accompanied by oxidative damage [27]. The fluorescence intensities of dityrosine, *N′*-formylkynurenine and kynurenine were monitored as markers of the oxidative changes in tyrosine and tryptophan amino acid residues from the incubations of BSA + MGO, in the presence of EEVc, brickellin and cordialin A. The fluorescence intensities relative to dityrosine, *N′*-formylkynurenine and kynurenine formation had progressive increases in the incubations of BSA + MGO when compared to BSA alone. BSA + MGO + AG decreased the formation of these amino acid oxidation markers, showing the responsiveness of this in vitro model system of protein glycation to interventions able to inhibit the protein oxidation due to glycation process (Figure 4). It can also be noted that none of the tested samples (EEVc, brickellin and cordialin A) with BSA promoted amino acid oxidative damage as much as BSA + MGO (Figures S14–S16 Supplementary Material).

In Figure 4A_I_–C_I_, the formation of dityrosine caused by the interaction BSA + MGO was increased and progressive over the days of the experiment. In Figure 4A_I_, it can be observed that the EEVc, at all tested concentrations, protected BSA against the formation of the dityrosine due to the incubation with MGO. In Figure 4B_I_, the incubation with brickellin was also efficient in reducing the formation of dityrosine. The incubation with cordialin A (Figure 4C_I_) attenuated the dityrosine formation with a concentration-dependent response; however, on days 4 and 8 there was no difference with BSA + MGO.

Dityrosine can be found as a product of protein degradation, and endogenous and exogenous agents, such as ultraviolet radiation, exposure to free radicals, lipid hydroperoxides and aging, can lead to dityrosine formation. Dityrosine has been used as a specific marker for protein oxidation and, consequently, to measure oxidative stress [62].

The formation of *N′*-formylkynurenine (Figure 4 A_II_) was decreased in the incubations of BSA + MGO in the presence of EEVc, which demonstrated a concentration-dependent response in all of the concentrations and days of experiment, with the exception of day 2, in which the EEVc (250 µg/mL) and AG were statistically equal, as were EEVc 125 and 62.5 µg/mL on the same day. In Figure 4B_II_, by day 2, the brickellin (0.125 mM) and AG protected the BSA against *N′*-formylkynurenine formation in the same way. Cordialin A had concentration-dependent effect on the inhibition of *N′*-formylkynurenine formation (Figure 4C_II_).

In vivo, *N′*-formylkynurenine and kynurenine are formed from the oxidation of tryptophan, a process that depends on the presence of reactive oxygen species and enzymes such as 2,3-dioxygenase and indoleamine-2,3-dioxygenase [63]. In the present study, both EEVc and brickellin at the highest concentration, in the first 2 days of incubation, prevented the formation of *N′*-formylkynurenine, which may be associated with the synergism with the antioxidant activity of EEVc and the fraction enriched with flavonoid compounds [6,7,9]. The oxidative degradation of Amadori protein-product intermediates causes the modification of protein tryptophan residues by oxidation via the hydroxyl radical, affecting their function under physiologically relevant conditions [64].

For kynurenine, BSA + MGO increased the formation of this oxidation marker (Figure 4). In the incubations of BSA + MGO with EEVc (Figure 4A_III_) or cordialin A (Figure 4C_III_), the inhibition of kynurenine formation occurred in a concentration-dependent response. On day 8, brickellin (Figure 4B_III_) at a concentration of 50 µg/mL inhibited the kynurenine formation, as well as AG.

In clinical therapy, tryptophan catabolism is relevant because of the metabolites that are generated, such as 3-hydroxyanthranilic acid, anthranilic acid and quinolic acid. These metabolites are associated with various neurological diseases and disorders, such as Alzheimer’s disease, Parkinson’s disease and amyotrophic lateral sclerosis [65,66], atherosclerosis [67], as well as cataract formation and the suppression of the proliferation of immune cells, such as T cells [68].

#### 3.2.2. Quantification of Carbonyl Groups in Proteins

Protein glycation and the process leading to the formation of AGEs result in the generation of highly reactive intermediates, such as dicarbonyl compounds and reactive oxygen species (ROS). The increased generation of ROS in the organism and the consequent inefficient action of the endogenous antioxidant mechanisms cause oxidative stress. The accumulation of ROS under conditions of oxidative stress induces lipid peroxidation and glycoxidation reactions, which exacerbates the formation of AGEs and ROS, intensifying the oxidative damage [69]. Research has shown that inflammatory diseases of chronic nature and neurodegenerative diseases have increases in the levels of carbonyl groups in proteins (PCO) in common. The products formed in the protein carbonylation are chemically stable, which favors their accumulation and detection in the body [70].

The PCO levels were monitored in the incubation samples on the last day of experiment, day 8 (Figure S17 Supplementary Material). The incubations of BSA + MGO exhibited the maximum response in the PCO formation, while the AG incubated with BSA + MGO had a mild effect on inhibiting the protein carbonylation, as already expected according to Colzani et al. [71]. Among the investigated samples, none of them caused the carbonylation of BSA when incubated in the absence of MGO. When incubated with BSA + MGO, EEVc and brickellin, at all tested concentrations, did not inhibit the PCO formation as much as AG. On the other hand, cordialin A exhibited the best effect on the inhibition of PCO formation, in a concentration-dependent response, with cordialin 0.051 and 0.025 mM being more effective than AG.

In vivo, moderately levels of PCO can be degraded by two main proteolytic pathways: the proteasomal and the autophagic/lysosomal systems [72]. However, proteins that are strongly carbonylated tend to form high molecular weight aggregates that are resistant to degradation, which favors their accumulation in the body [73], a condition that is age-dependent [72].

The low efficiency of EEVc and brickellin to inhibit PCO formation suggested that they are more effective in directly stabilizing dicarbonyl compounds, such as MGO (considering the best results on inhibiting AGE formation), than in decreasing the oxidative damage to BSA damage generated by the exposure to MGO. The performance of cordialin A on PCO formation inhibition was different from the other samples, in that the higher the concentration, the lower the PCO levels. The data in the literature show that the efficiency of triterpenes on inhibiting the formation of AGEs occurs more broadly and may be due to: (a) the interaction with macromolecules involved in the glycation process [54,61], either by modulating the activity and/or expression of enzymes; (b) by facilitating the metabolization of AGEs [59,60]; or (c) by the ability to scavenge free radicals [58]. So far, the results of our study suggest that cordialin A may be interacting with BSA, decreasing the damage caused by MGO; however, this hypothesis still needs to be further investigated.

#### 3.2.3. Modification of BSA via Crosslinking

**Figure 5:**
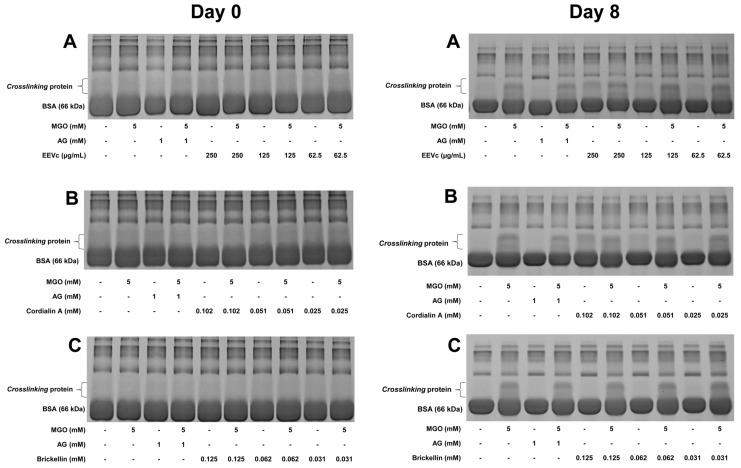
Effects of (**A**) EEVc, (**B**) cordialin A and (**C**) brickellin on protein crosslinking formation on day 0 and day 8 in an in vitro protein glycation model system using BSA and MGO. BSA: bovine serum albumin; MGO: methylglyoxal; AG: aminoguanidine; EEVc: ethanolic extract of *V. curassavica*.

Detrimental effects occur when crosslinks are formed in proteins, which represent the major late consequences of protein glycoxidation. Protein crosslinking is formed from the interaction of dicarbonyl compounds with amino acid residues present in proteins and/or due to rearrangements of Amadori products [74,75]. This condition is accelerated by the presence of free radicals, leading to the cleavage and production of protein fragments and, consequently, cause impairments in their physical, chemical and functional properties [76,77]. Studies have shown how the interaction of glycated albumin with drugs impacts their pharmacokinetics, such as anti-inflammatory drugs [78,79,80,81,82]. Among the methods used for protein crosslink determination, electrophoresis is one of the most common [26,31,83]. The analysis of the protein crosslinking was performed with the incubation samples from day 0 and day 8 (Figure 5). The samples evaluated were EEVc at concentrations of 250, 125 and 62.5 µg/mL; brickellin 0.125; 0.062; and 0.031 mM and cordialin A 0.102, 0.051 and 0.025 mM, with AG (1 mM) as control (Figure 5A–C on day 0).

As previously stated, the formation of crosslinking causes structural damage to the proteins and, thus, with electrophoresis, it is possible to visualize a band above that corresponding to BSA (66 kDa). With this, the relative density generated by this new band is directly related to the oxidative damage suffered by BSA [56]. The densitometry values generated in the bands corresponding to crosslinking were also calculated (Figure S18 Supplementary Material).

Figure 5 also shows the data obtained on day 0 and day 8. On day 0, it is possible to note that, visually, none of the tested samples interacted with the BSA in a relevant manner that resulted in the formation of protein crosslink. On day 8, the last day of analysis, the formation of protein crosslinking in BSA + MGO was noticeable, a process that did not occur in BSA incubated alone and in the presence of AG on the same day of analysis.

The densitometric data of the incubation of BSA + AG and BSA alone were similar; therefore, as reported in the literature, the AG does not contribute to the formation of crosslinking. The same was observed with BSA + brickellin at concentrations of 0.125 and 0.031 mM, which were the same as the BSA + AG on day 8 (Figure 5C on day 8); however, the opposite happened with cordialin A (Figure 5B on day 8). The crosslinking promoted by cordialin A was directly proportional to its concentration, where the concentration of cordialin A 0.102 mM promoted greater crosslinking in BSA than cordialin A 0.025 mM incubated with BSA alone, which reinforces the hypothesis that cordialin A interacts with BSA, influencing the damage caused by the exposure to MGO. In general, the best performance was observed with brickellin, where all of the tested concentrations generated the minor damage to the BSA. The EEVc also promoted the formation of protein crosslinking (Figure 5A on day 8), which can be justified by the fact that the extract contains cordialin A and other compounds still unknown in its composition; however, it still had a satisfactory performance.

## 4. Conclusions

The ethanolic extract of *V. curassavica* leaves (EEVc) and the two compounds isolated from EEVc (brickellin and cordialin A) showed to be potent candidates against the inhibition of AGE formation by reducing the oxidative damage and its deleterious effects. It should be noted that there was no significant difference between the inhibition promoted by brickellin and EEVc at the highest concentrations (0.0125 mM and 250 µg/mL) within the first hours of the experiment, while cordialin A (0.102 mM), with a concentration-dependent response, promoted protein crosslinking when incubated with albumin, which may explain the slight crosslink formation in the presence of EEVc. The results found in the literature suggest that the antioxidant activity of EEVc and brickellin favor directly MGO. However, in vivo studies and kinetic analysis of the adduct formation may explain this mechanism of action.

## Figures and Tables

**Table 1:** NMR spectrometric data for brickellin (2′,5-dihydroxy-4′,3,5′,6,7-pentamethoxyflavone) at 400 MHz for ^1^H and 100 MHz for ^13^C in CDCl_3_. Data from the literature are also presented.

C	*δ* ¹³C	*δ* ^1^H *J*	*δ* ¹³C ^a^	*δ* ^1^H *J* ^a^	*δ* ^1^H *J* ^b^	*δ* ¹³C ^c^	*δ* ^1^H *J* ^c^
2	155.9 *s*	----	155.5 *s*	----	----	155.4 *s*	----
3	136.6 *s*	----	136.3 *s*	----	----	140.4 *s*	----
4	177.4 *s*	----	176.9 *s*	----	----	179.0 *s*	----
5	152.8 *s*	----	152.3 *s*	----	----	153.0 *s*	----
6	132.6 *s*	----	132.0 *s*	----	----	132.6 *s*	----
7	159.1 *s*	----	158.5 *s*	----	----	158.7 *s*	----
8	90.6 *d*	6.52 *s*	90.4 *d*	6.35 *s*	6.62 *s*	90.6 *d*	6.67 *s*
9	152.8 *s*	----	153.4 *s*	----	----	155.4 *s*	----
10	106.4 *s*	----	105.9 *s*	----	----	113.1 *s*	----
1′	108.7 *s*	----	108.2 *s*	----	----	107.1 *s*	----
2′	154.0 *s*	----	152.3 *s*	----	----	152.9 *s*	----
3′	103.0 *d*	6.64 *s*	102.6 *d*	6.45 *s*	6.59 *s*	99.9 *d*	6.43 *s*
4′	151.3 *s*	----	150.8 *s*	----	----	152.9 *s*	----
5′	143.6 *s*	----	143.1 *s*	----	----	140.4 *s*	----
6′	111.2 *d*	7.11 *s*	110.9 *d*	6.88 *s*	7.10 *s*	110.2 *d*	6.94 *s*
OMe	62.1 *q*	3.89 *s*	61.7 *q*	3.70–4.00 *s*	3.80 *s*	60.9 *q*	3.79 *s*
OMe	60.8 *q*	3.93 *s*	60.5 *q*	3.70–4.00 *s*	3.90 *s*	60.6 *q*	3.80 *s*
OMe	56.0 *q*	3.97 *s*	56.5 *q*	3.70–4.00 *s*	3.93 *s*	56.3 *q*	3.91 *s*
OMe	56.4 *q*	3.94 *s*	56.1 *q*	3.70–4.00 *s*	3.93 *s*	56.4 *q*	3.91 *s*
OMe	56.8 *q*	3.93 *s*	55.7 *q*	3.70–4.00 *s*	3.96 *s*	56.8 *q*	3.91 *s*
2′-OH	----	7.90 *s*	----	----	7.87 *s*	----	----
5-OH	----	12.38 *s*	----	----	12.36 *s*	----	----

(^a^) ROBERTS et al. [33]: brickellin ^13^C NMR data in CDCl_3_ and ^1^H in CCl_4_. (^b^) IINUMA et al. [34]: brickellin ^1^H NMR data in CDCl_3_. (^c^) IINUMA et al. [34]: 4′,5-dihydroxy-2′,3,5′,6,7-pentamethoxyflavone ^13^C and ^1^H NMR data in CDCl_3_.

**Table 2:** NMR spectrometric data for cordialin A at 400 MHz for ^1^H and 100 MHz for ^13^C in CDCl_3_. Data from the literature are also presented.

C	*δ* ¹³C	*δ* ^1^H *J* (Hz)	*δ* ¹³C ^a^	*δ* ^1^H *J* (Hz) ^a^	*δ* ¹³C ^b^	*δ* ^1^H *J* (Hz) ^b^
1	30.0 *t*	3.12 *td* (12.4; 5.9)	29.8 *t*	3.12 *td* (12.5; 6.0)	30.0 *t*	3.11 *td* (12.7; 5.9)
2	37.8 *t*	nd *	37.8 *t*	nd	37.9 *t*	nd
3	98.4 *s*	-----	98.7 *s*	----	98.6 *s*	----
4	36.1 *s*	-----	36.1 *s*	----	36.1 *s*	----
5	51.0 *d*	1.31 *dl* (2.8)	50.9 *d*	nd	50.9 *d*	1.31 *dl* (3.3)
6	19.4 *t*	1.66 *m* 1.48 *m*	19.4 *t*	nd	19.4 *t*	1.67 *dd* (12.3; 3.3) 1.48 *m*
7	34.3 *t*	nd	34.3 *t*	nd	34.4 *t*	nd
8	39.9 *s*	----	39.9 *s*	----	39.9 *s*	----
9	50.1 *d*	1.57 *d* (10.8)	50.0 *d*	nd	50.1 *d*	1.56 *d* (11.0)
10	40.9 *s*	nd	41.0 *s*	nd	41.0 *s*	nd
11	70.9 *d*	3.67 (10.8; 4.0)	70.5 *d*	3.65 *td* (10.5; 4.2)	70.8 *d*	3.65 *td* (11.0; 3.9)
12	37.2 *t*	1.89 *m*	36.9 *t*	nd	37.2 *t*	1.85 *td* (11.2; 3.9)
13	44.6 *d*	nd	44.8 *d*	nd	44.7 *d*	nd
14	49.0 *s*	nd	49.0 *s*	----	49.0 *s*	----
15	31.6 *t*	----	31.7 *t*	nd	31.7 *t*	nd
16	27.8 *t*	nd	27.9 *t*	nd	27.8 *t*	nd
17	51.3 *d*	2.38 *td* (10.4; 6.0)	51.5 *d*	2.40 *td* (11.0; 6.0)	51.4 *d*	2.38 *td* (10.5; 5.7)
18	15.4 *q*	0.95 *s*	15.4 *q*	0.95 *s*	15.4 *q*	0.95 *s*
19	67.8 *t*	4.16 *dd* (8.6; 1.4) 4.33 *dd* (8.6; 2.5)	67.8 *t*	*α*-H 4.15 *dd* (8.5; 0.7) *β*-H 4.32 *dd* (8.5; 2.0)	67.9 *t*	4.15 *dd* (8.5;1.0) 4.31 *dd* (8.5; 2.5)
20	164.3 *s*	----	164.8 *s*	-----	164.6 *s*	----
21	17.2 *q*	2.12 *d* (0.8)	17.3 *q*	2.12 *sl*	17.3 *q*	2.11 *sl*
22	120.3 *d*	6.28 *sl*	120.4 *d*	6.28 *sl*	120.5 *d*	6.27 *sl*
23	195.7 *s*	----	195.7 *s*	----	195.7 *s*	----
24	66.3 *d*	3.34 *s*	66.4 *d*	3.34 *s*	66.4 *d*	3.33 *s*
25	61.0 *s*	----	61.2 *s*	----	61.2 *d*	----
26	24.9 *q*	1.43 *s*	24.9 *q*	1.42 *s*	25.0 *d*	1.42 *s*
27	18.7 *q*	1.27 *s*	18.6 *q*	1.27 *s*	18.7 *q*	1.26 *s*
28	26.3 *q*	1.04 *s*	26.4 *q*	1.03 *s*	26.4 *q*	1.03 *s*
29	18.9 *q*	1.01 *s*	18.8 *q*	1.01 *s*	18.9 *q*	1.00 *s*
30	16.8 *q*	0.91 *s*	16.8 *q*	0.90 *s*	16.9 *q*	0.90 *s*

(**a**) VELDE et al. [36]: cordialin A ^13^C and ^1^H NMR data in CDCl_3_. (**b**) PEREIRA et al. [32]: cordialin A ^13^C and ^1^H NMR data in CDCl_3_. * nd= not detected.

## Data Availability

Data are contained within the article.

## Supplementary Materials

The following supporting information can be downloaded at: https://www.mdpi.com/article/10.3390/antiox12020522/s1, Figure S1: Chromatoplates (A and B) and chromatograms (C) of EEVc and its fractions; Figure S2: Spectrometric data of brickellin; Figure S3: 1H NMR spectrum of brickellin obtained at 400 MHz in CDCl3 (20 mg/mL); Figure S4: 13C NMR spectrum of brickellin obtained at 75 MHz in CDCl3 (20 mg/mL); Figure S5: Spectrometric data of cordialin A; Figure S6: ¹H NMR spectrum of cordialin A obtained at 400 MHz in CDCl3 (20 mg/mL); Figure S7: Expansions of the ¹H NMR spectrum of cordialin A obtained at 400 MHz in CDCl3 (20 mg/mL); Figure S8: 13C NMR spectrum of cordialin A obtained at 100 MHz in CDCl3 (20 mg/mL); Figure S9: Effects of different concentrations of EEVc (A), brickellin (B) and cordialin A (C) on the formation of fluorescent AGEs in vitro protein glycation model system with BSA alone; Figure S10: Absorbance plots of the controls used in the in vitro protein glycation model system; Figure S11: Absorbance plots of EEVc 250; 125 and 62.5 µg/mL used in the in vitro protein glycation model system; Figure S12: Absorbance plots of brickellin 0.125; 0.062; and 0.031 mM used in the in vitro protein glycation model system; Figure S13: Absorbance plots of cordialin A 0.102; 0.051; and 0.025 mM used in the in vitro protein glycation model system; Figure S14: Effects of different concentrations of EEVc (A), brickellin (B) and cordialin A (C) on dityrosine formation in BSA-only protein glycation model system in vitro; Figure S15: Effects of different concentrations of EEVc (A), brickellin (B) and cordialin A (C) on N’-formylkynurenine formation in BSA-only protein glycation model system in vitro; Figure S16: Effects of different concentrations of EEVc (A), brickellin (B) and cordialin A (C) on the formation of Quinurenin in BSA-only protein glycation model system in vitro; Figure S17: Quantification of carbonylated proteins obtained on day 8 in vitro protein glycation model system using BSA and MGO; Figure S18: Graphical representation of densitometry calculation generated by ImageJ^®^ 1.53k program regarding crosslinking formation of EEVc, brickellin and cordialin A samples at different concentrations incubated with BSA+MGO on day 8.
