# Supplementary material for: In Vitro Antiglycation Potential of Erva-Baleeira (Varronia curassavica Jacq.)
Source: Antioxidants (Basel). 2023 Feb 19;12(2):522. doi: 10.3390/antiox12020522 (PMC9952575; doi:10.3390/antiox12020522)
Supplement: Supplementary file 1 [file antioxidants-12-00522-s001.zip › antioxidants-2202056-supplementary.pdf]

SUPPLEMENTARY MATERIAL

In vitro antiglycation potential of erva-baleeira (*Varronia curassavica* Jacq.)

Winner Duque Rodrigues[1], Felipe Nunes Cardoso[2], Amanda Martins Baviera[2],* and André Gonzaga dos Santos[1],*

[1]Department of Drugs and Medicines, School of Pharmaceutical Sciences, São Paulo State University
[2]Department of Clinical Analysis, School of Pharmaceutical Sciences, São Paulo State University

*Correspondence: andre.gonzaga@unesp.br (AGS); amanda.baviera@unesp.br (AMB), zip code: 14800-903 - Araraquara, SP, Brazil

Abstract: Background: *Varronia curassavica* Jacq. is traditionally used in the treatment of inflammatory processes. The ethanolic extract of its leaves (EEVc) showed anti-inflammatory and low toxicity. Medicinal plants aroused interest for antiglycation activity. The formation and accumulation of advanced glycation end products (AGEs) is associated with several chronic diseases. The objective of this study was to evaluate the antiglycation potential of the EEVc and two isolated compounds. Methods: The compounds brickellin and cordialin A were obtained by chromatographic methods and identified by spectrometric techniques. Analyses of fluorescent AGEs, biomarkers of amino acid residue oxidation, protein carbonylated levels and crosslinking formation were performed in samples obtained from an *in vitro* model system of protein glycation with methylglyoxal. Results: EEVc, brickellin and cordialin A significantly reduced the in vitro formation of AGEs, and reduced the damage caused by oxidative damage to the protein. Conclusions: According to the results EEVc, brickellin and cordialin A are potential candidates against AGEs formation, which opens the way to expand the therapeutic arsenal for many pathologies that AGEs are involved.

Keywords: Cordia verbenacea; Antiglycation activity; Oxidative stress; Cordialin A; Brickellin.

List of figures

Figure S1: Chromatoplates (A and B) and chromatograms (C) of EEVc and its fractions. 4

Figure S2: Spectrometric data of brickellin........ 4

Figure S3: ^{1}H NMR spectrum of brickellin obtained at 400 MHz in $CDCl_3$ (20 mg/mL). 5

Figure S4: ^{13}C NMR spectrum of brickellin obtained at 75 MHz in $CDCl_3$ (20 mg/mL).5

Figure S5: Spectrometric data of cordialin A........ 6

Figure S6: ^{1}H NMR spectrum of cordialin A obtained at 400 MHz in $CDCl_3$ (20 mg/mL). 7

Figure S7: Expansions of the ^{1}H NMR spectrum of cordialin A obtained at 400 MHz in $CDCl_3$ (20 mg/mL)........ 7

Figure S8: ^{13}C NMR spectrum of cordialin A obtained at 100 MHz in $CDCl_3$ (20 mg/mL). 8

Figure S9: Effects of different concentrations of EEVc (A), brickellin (B) and cordialin A (C) on the formation of fluorescent AGEs *in vitro* protein glycation model system with BSA alone........ 8

Figure S10: Absorbance plots of the controls used in the *in vitro* protein glycation model system........ 9

Figure S11: Absorbance plots of EEVc 250; 125 and 62.5 µg/mL used in the *in vitro* protein glycation model system........ 10

Figure S12: Absorbance plots of brickellin 0.125; 0.062; and 0.031 mM used in the *in vitro* protein glycation model system........ 11

Figure S13: Absorbance plots of cordialin A 0.102; 0.051; and 0.025 mM used in the *in vitro* protein glycation model system........ 12

Figure S14: Effects of different concentrations of EEVc (A), brickellin (B) and cordialin A (C) on dityrosine formation in BSA-only protein glycation model system *in vitro*... 13

Figure S15: Effects of different concentrations of EEVc (A), brickellin (B) and cordialin A (C) on *N'*-formylkynurenine formation in BSA-only protein glycation model system *in vitro*........ 13

Figure S16: Effects of different concentrations of EEVc (A), brickellin (B) and cordialin A (C) on the formation of Quinurenin in BSA-only protein glycation model system *in vitro*........ 14

Figure S17: Quantification of carbonylated proteins obtained on day 8 *in vitro* protein glycation model system using BSA and MGO........ 14

Figure S18: Graphical representation of densitometry calculation generated by ImageJ® 1.53k program regarding crosslinking formation of EEVc, brickellin and cordialin A samples at different concentrations incubated with BSA+MGO on day 8. 15

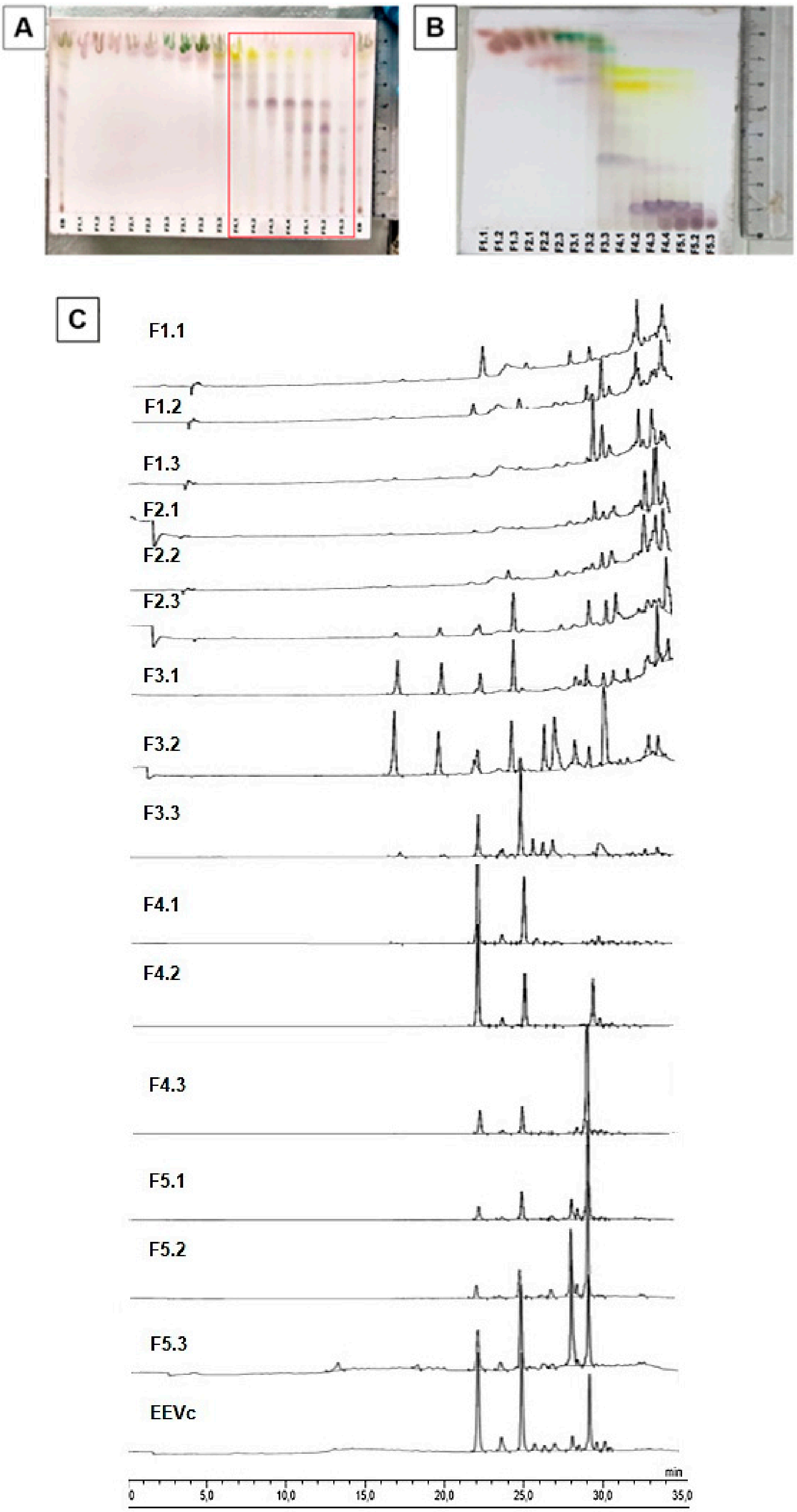
A
B
C
F1.1
F1.2
F1.3
F2.1
F2.2
F2.3
F3.1
F3.2
F3.3
F4.1
F4.2
F4.3
F5.1
F5.2
F5.3
EEVc
min
0
5,0
10,0
15,0
20,0
25,0
30,0
35,0

Figure S1: Chromatoplates (A and B) and chromatograms (C) of EEVc and its fractions. The fractions and the EEVc solutions (5 mg/mL, ethyl acetate) were applied (40 µL) in silica gel chromatoplates using as mobile phases: A) chloroform: ethyl acetate: methanol 5.5:3.5:1 (v/v); B) chloroform: ethyl acetate 6:4 (v/v). The spray reagent was 10% sulfuric acid in ethanol (110º C, 10 min). (C) After SPE in methanol: water (95:05), 20 µL of each sample (1.0 mg/mL, methanol) were analyzed on HPLC-UV with a C18 column under a linear gradient of 5-100% methanol in 30 min plus methanol in 5 min; flow rate of 1.0 mL/min; detector wavelength at 254 nm.

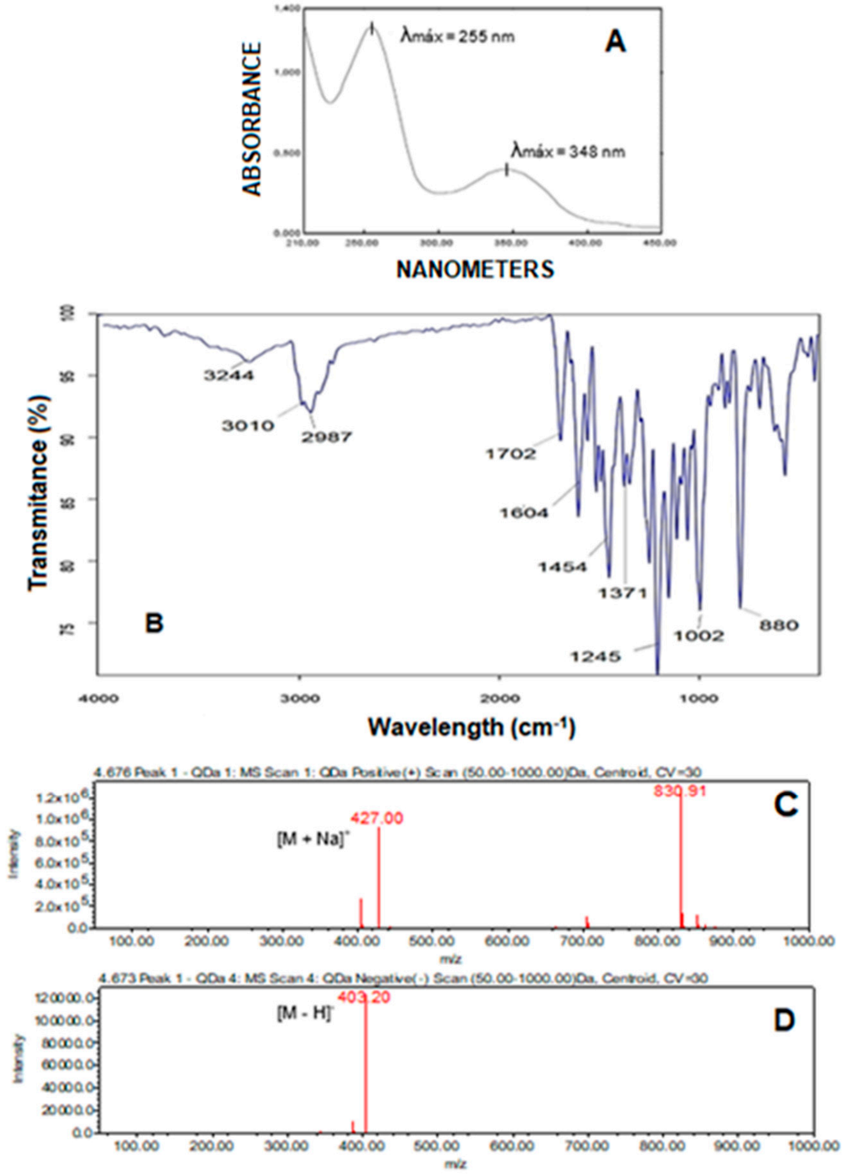

Figure S2: Spectrometric data of brickellin. (A) UV spectrum (210-450 nm), 1.0 mg/mL (methanol); (B) Fourier transform infrared absorption spectrum; (C) Mass spectra of brickellin by HPLC-MS in the positive mode (ESI$^+$), *m/z*= 427.00 $[M+Na]^+$ and (D) in the negative mode (ESI$^-$), *m/z*= 403.20 $[M-H]^-$; chromatographic conditions: C18 colum (250 x 4.6 mm, 5 µm), isocratic methanol: water acidified with 2% acetic acid (75:25) for 35 min, flow rate of 1.0 mL/min and injection volume of 20 µL.

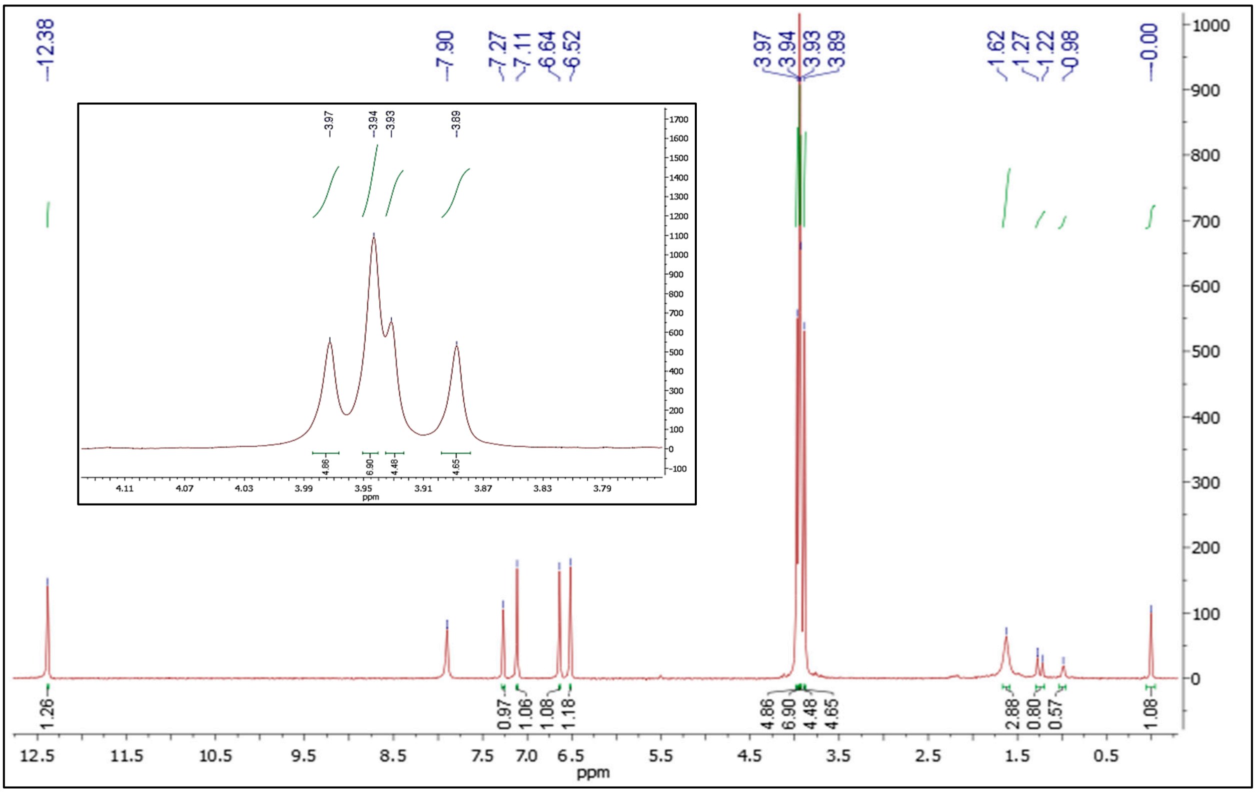

Figure S3: ^{1}H NMR spectrum of brickellin obtained at 400 MHz in $CDCl_3$ (20 mg/mL).

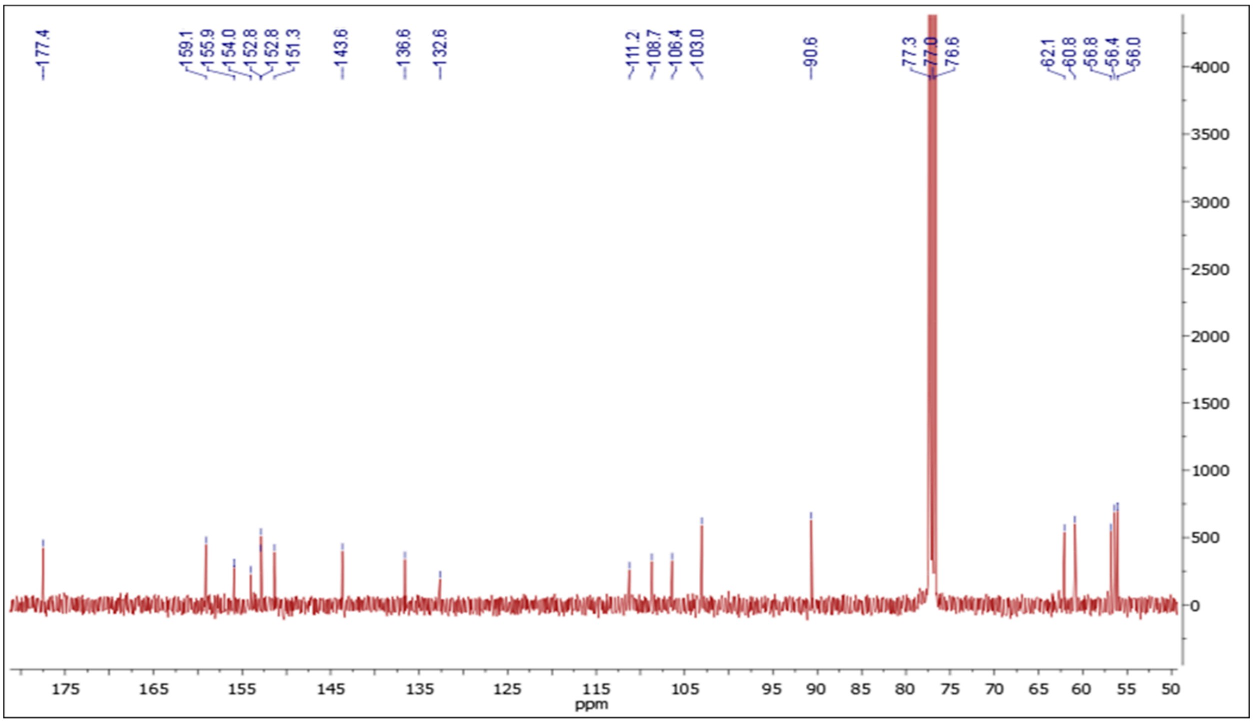

Figure S4: ^{13}C NMR spectrum of brickellin obtained at 75 MHz in $CDCl_3$ (20 mg/mL).

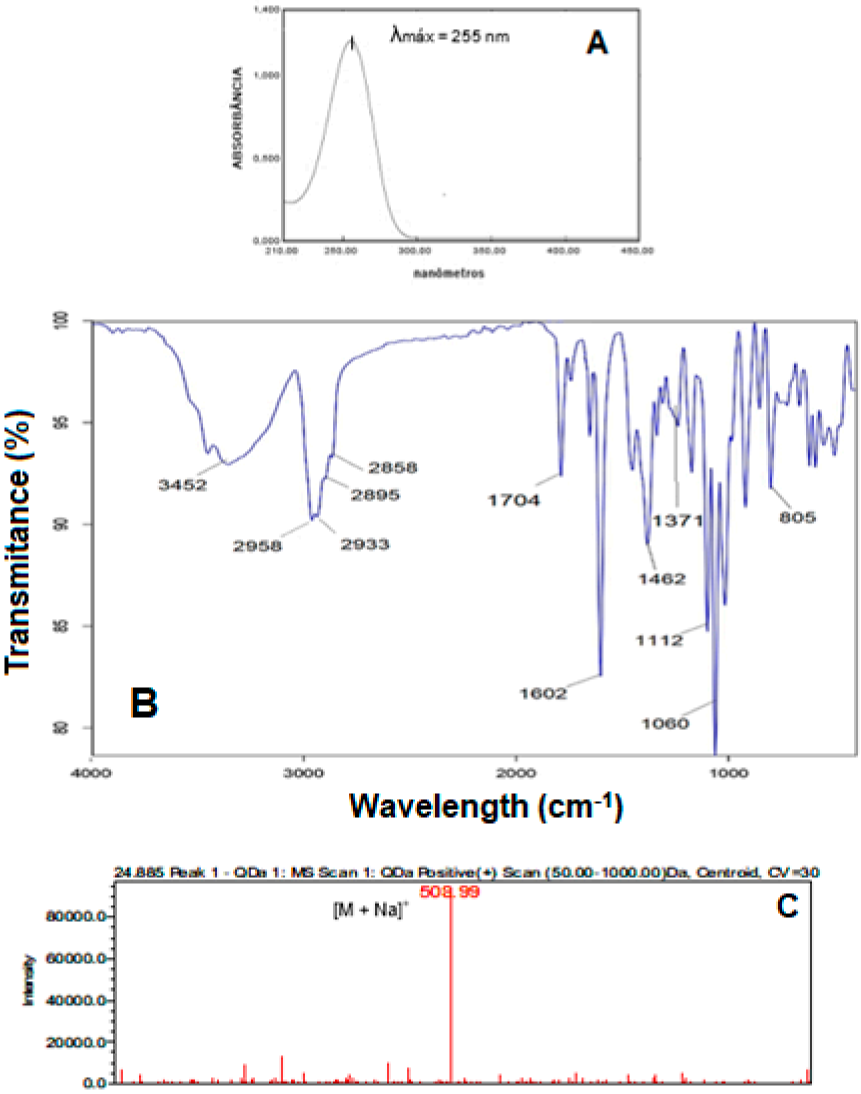

Figure S5: Spectrometric data of cordialin A. (A) UV spectrum (210-450 nm), 1.0 mg/mL (methanol); (B) Fourier transform infrared absorption spectrum; (C) Mass spectra of cordialin A by HPLC-MS in the positive mode (ESI$^+$), *m/z*= 508.99 $[M+Na]^+$; chromatographic conditions: C18 colum (250 x 4.6 mm, 5 µm), isocratic methanol: water acidified with 2% acetic acid (75:25) for 35 min, flow rate of 1.0 mL/min and injection volume of 20 µL.

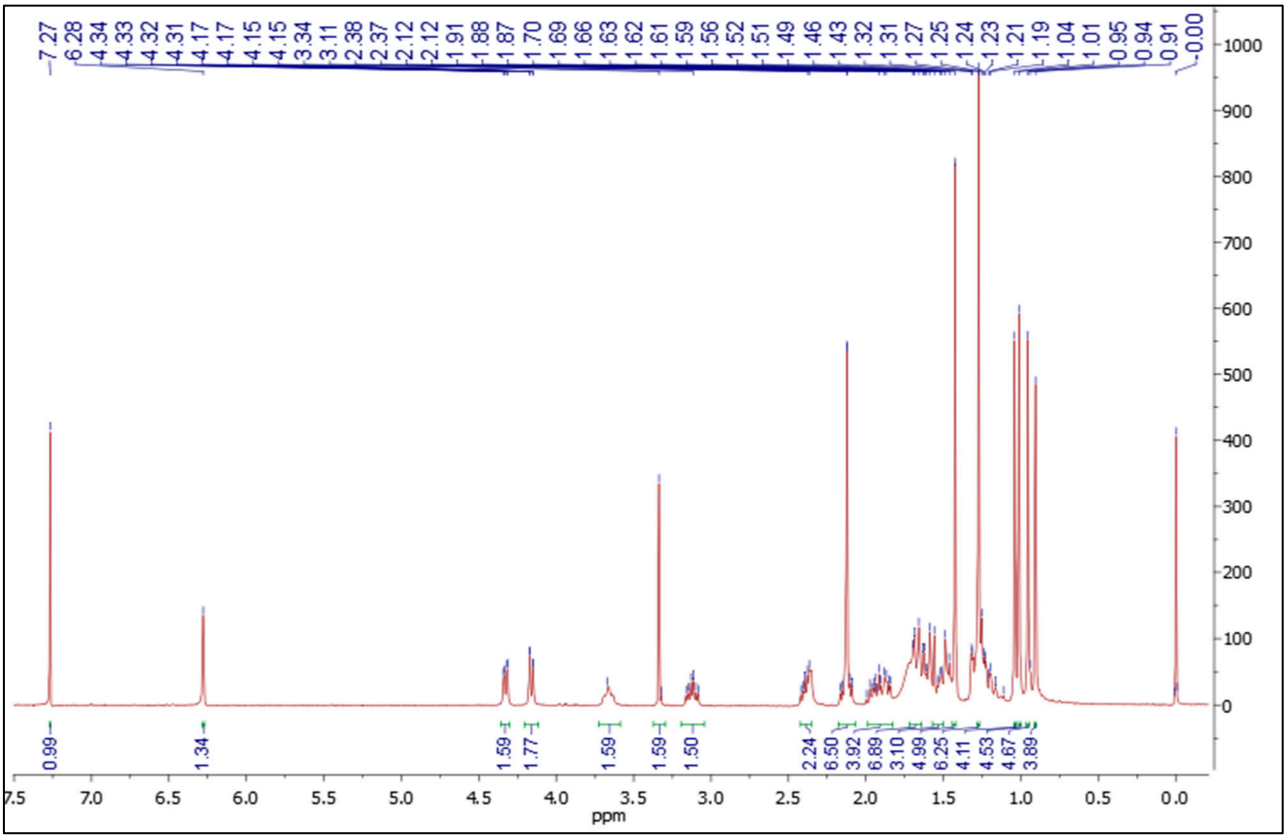

Figure S6: ^{1}H NMR spectrum of cordialin A obtained at 400 MHz in $CDCl_3$ (20 mg/mL).

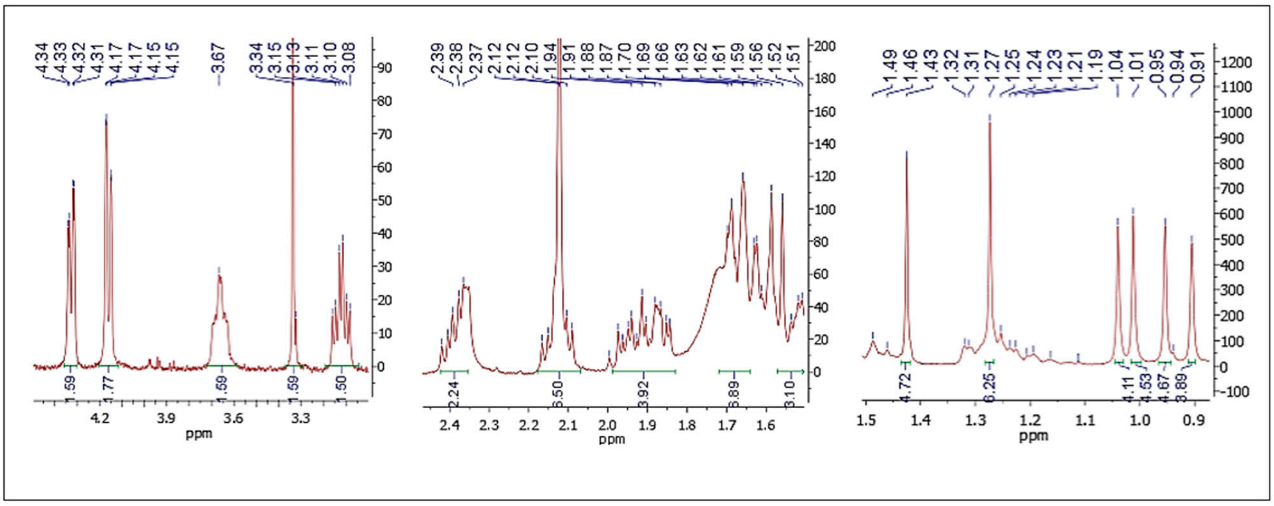

Figure S7: Expansions of the ^{1}H NMR spectrum of cordialin A obtained at 400 MHz in $CDCl_3$ (20 mg/mL).

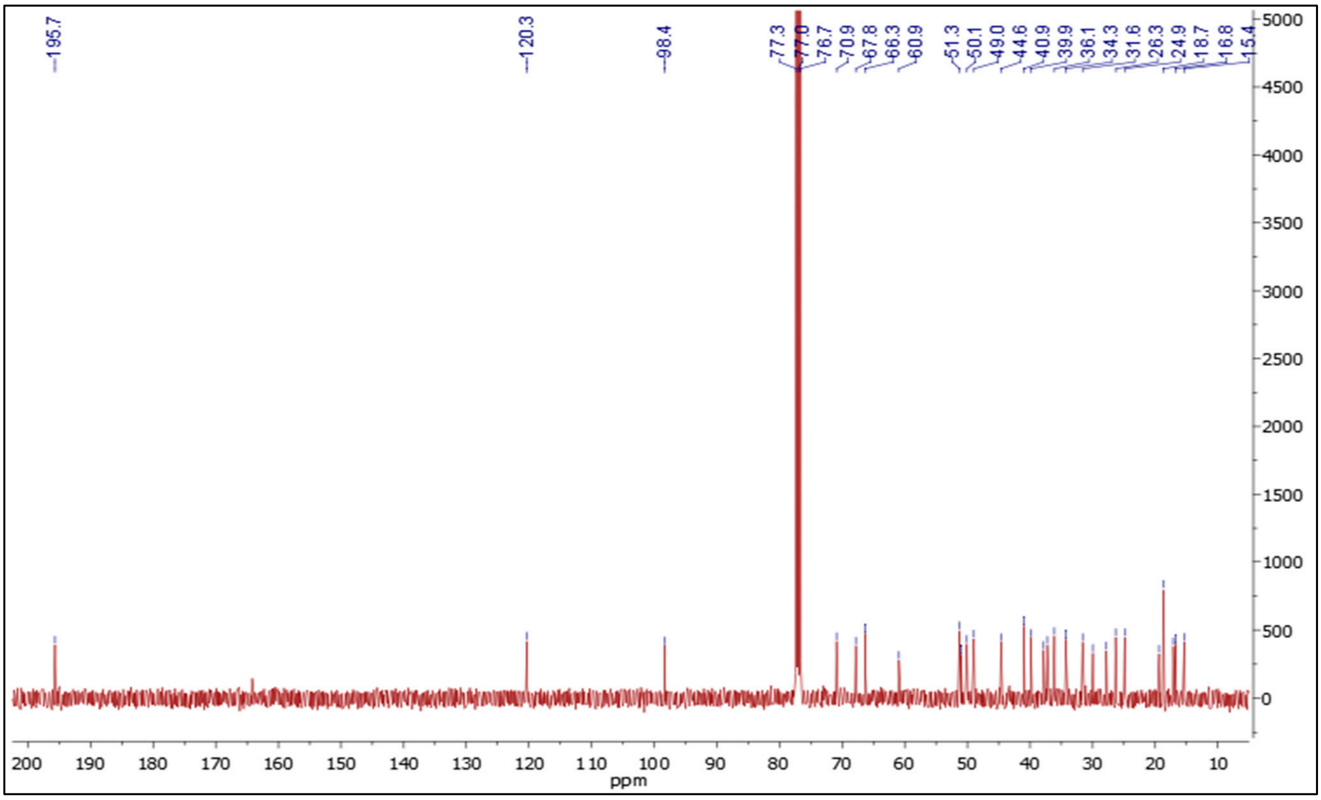

Figure S8: ^{13}C NMR spectrum of cordialin A obtained at 100 MHz in $CDCl_3$ (20 mg/mL).

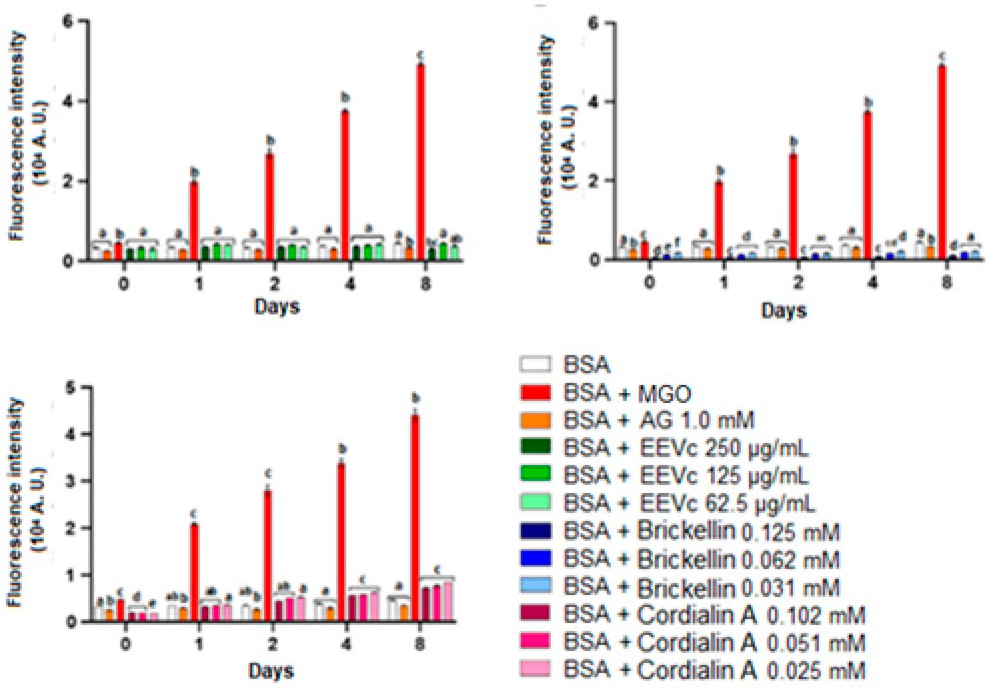

Figure S9: Effects of different concentrations of EEVc (A), brickellin (B) and cordialin A (C) on the formation of fluorescent AGEs *in vitro* protein glycation model system with BSA alone**.**

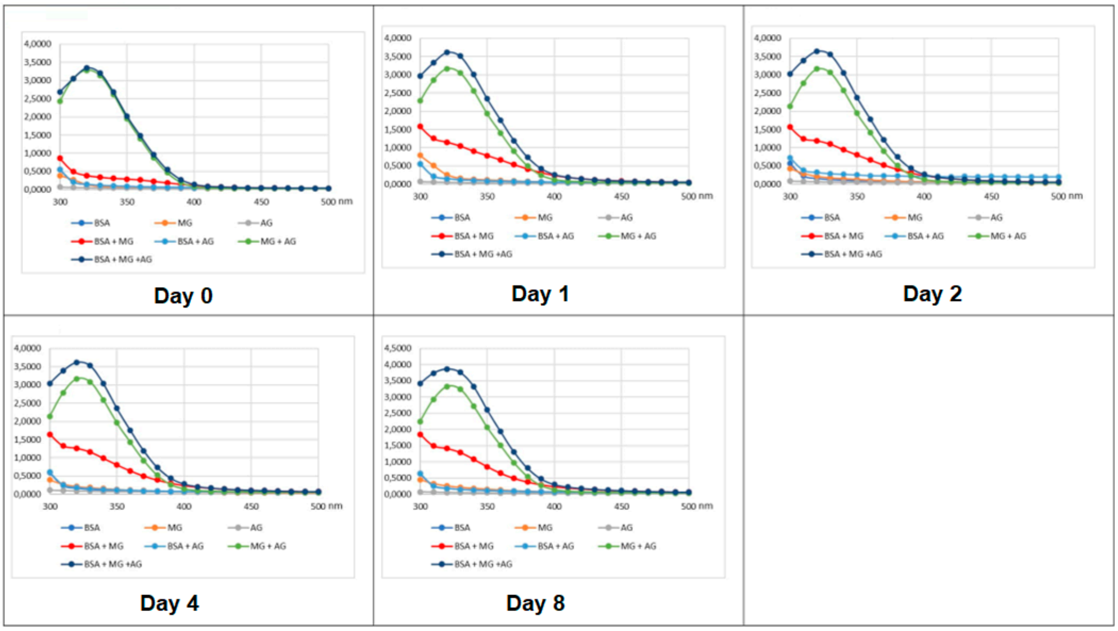

Figure S10: Absorbance plots of the controls used in the *in vitro* protein glycation model system.

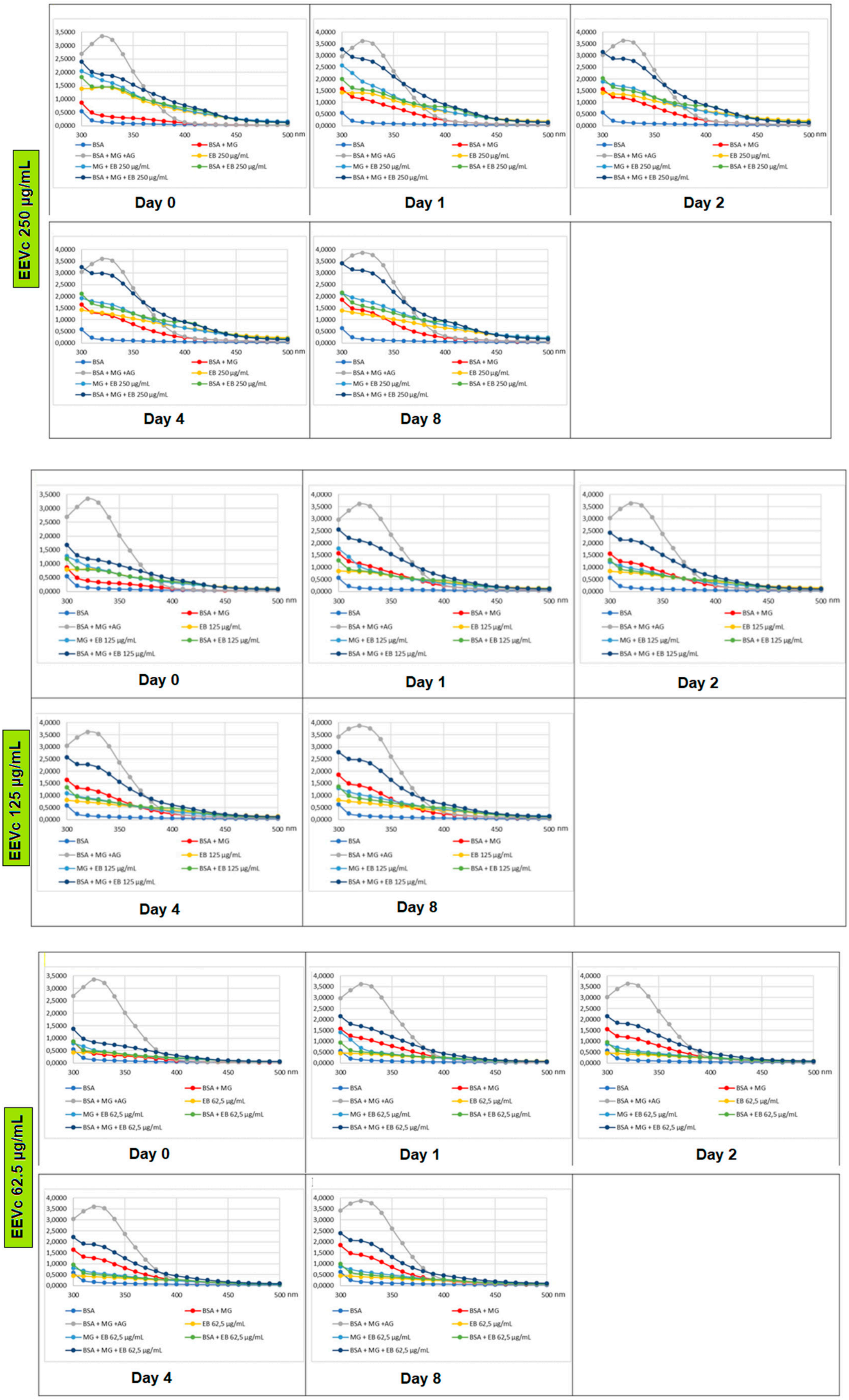

Figure S11: Absorbance plots of EEVc 250; 125 and 62.5 µg/mL used in the *in vitro* protein glycation model system.

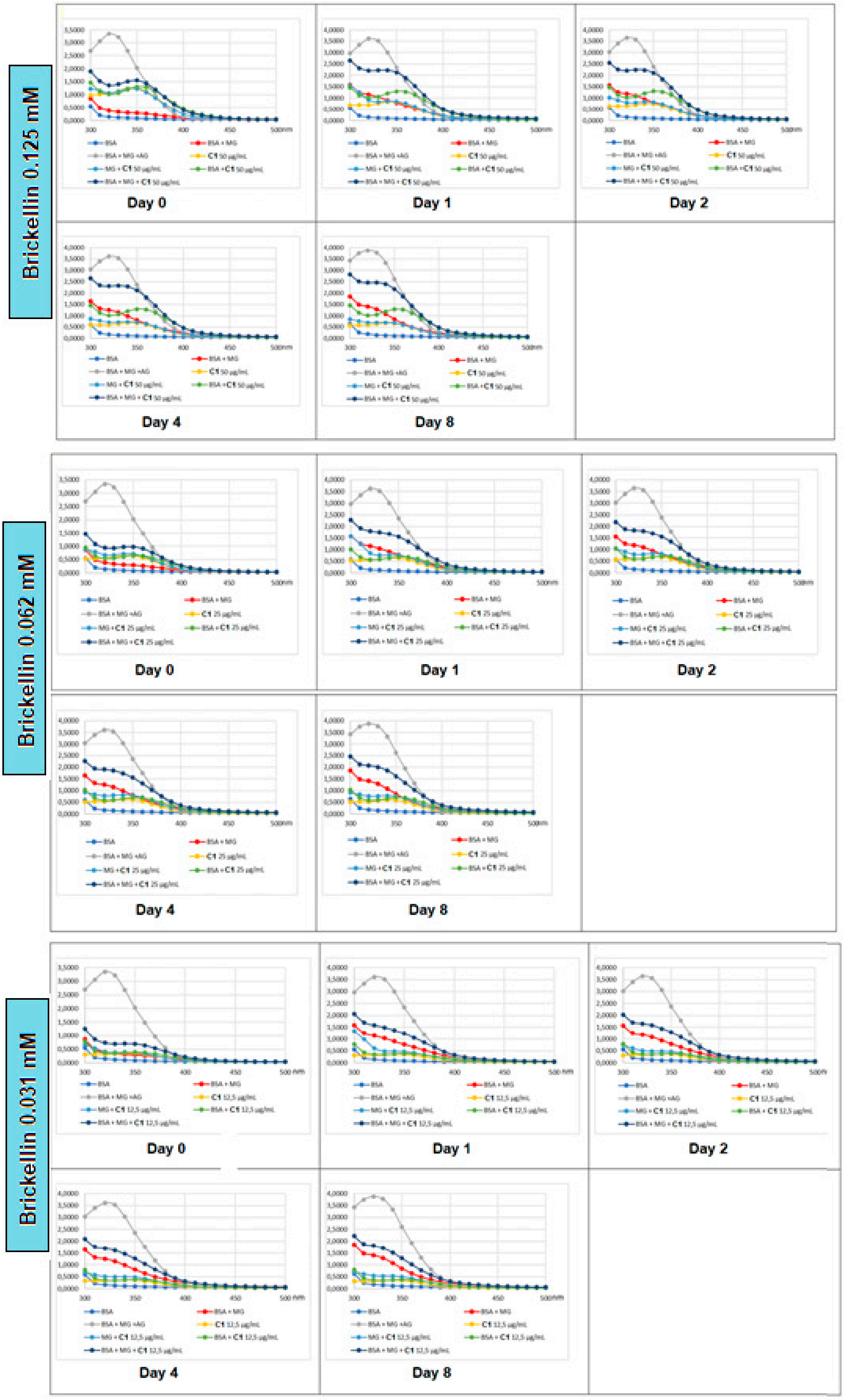

Figure S12: Absorbance plots of brickellin 0.125; 0.062; and 0.031 mM used in the *in vitro* protein glycation model system.

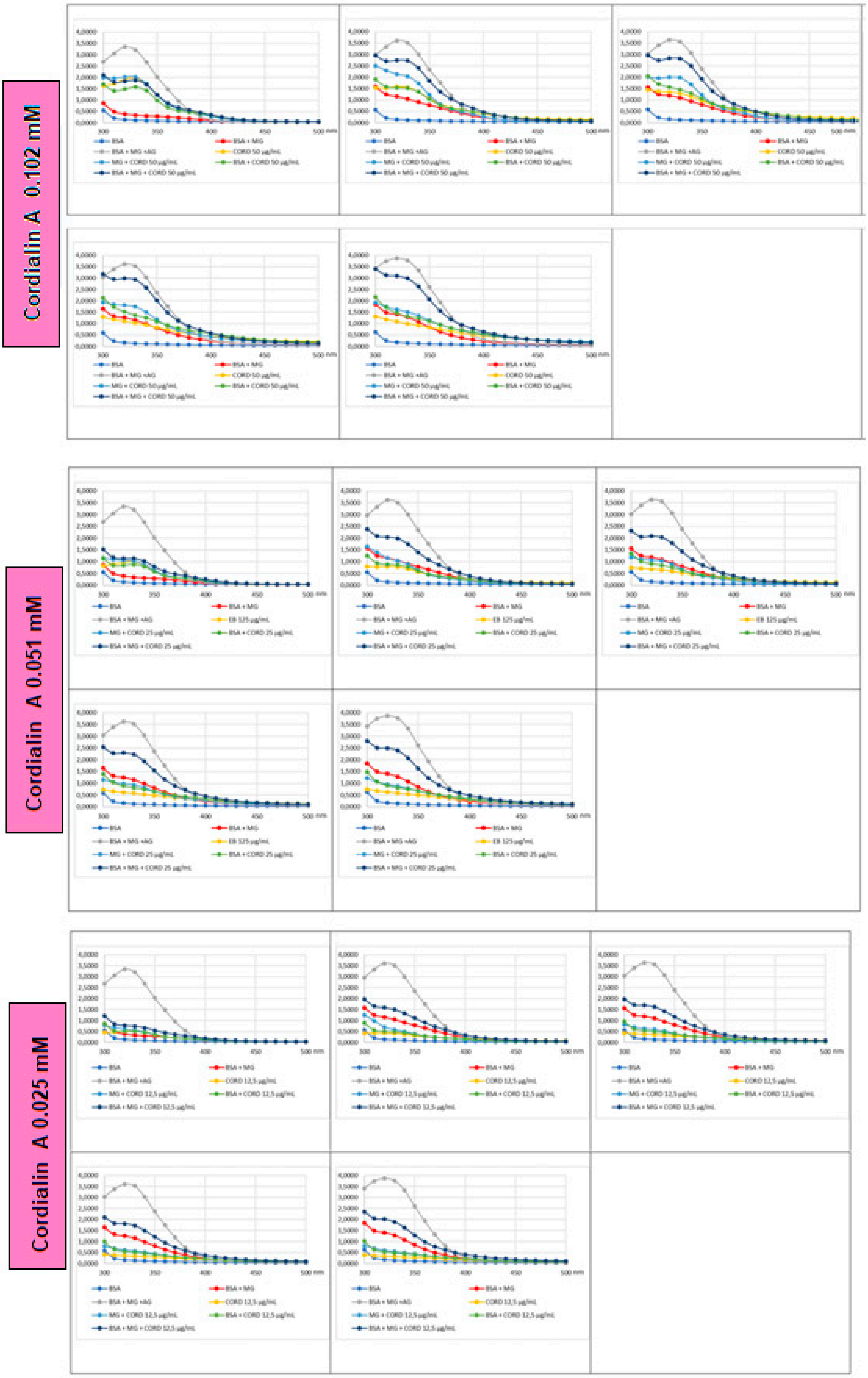

Figure S13: Absorbance plots of cordialin A 0.102; 0.051; and 0.025 mM used in the *in vitro* protein glycation model system.

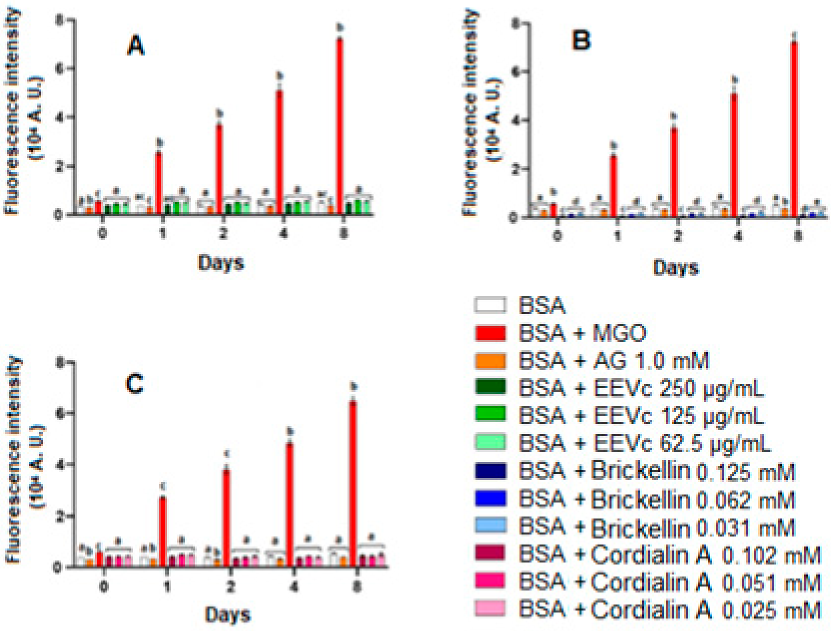

Figure S14: Effects of different concentrations of EEVc (A), brickellin (B) and cordialin A (C) on dityrosine formation in BSA-only protein glycation model system *in vitro*.

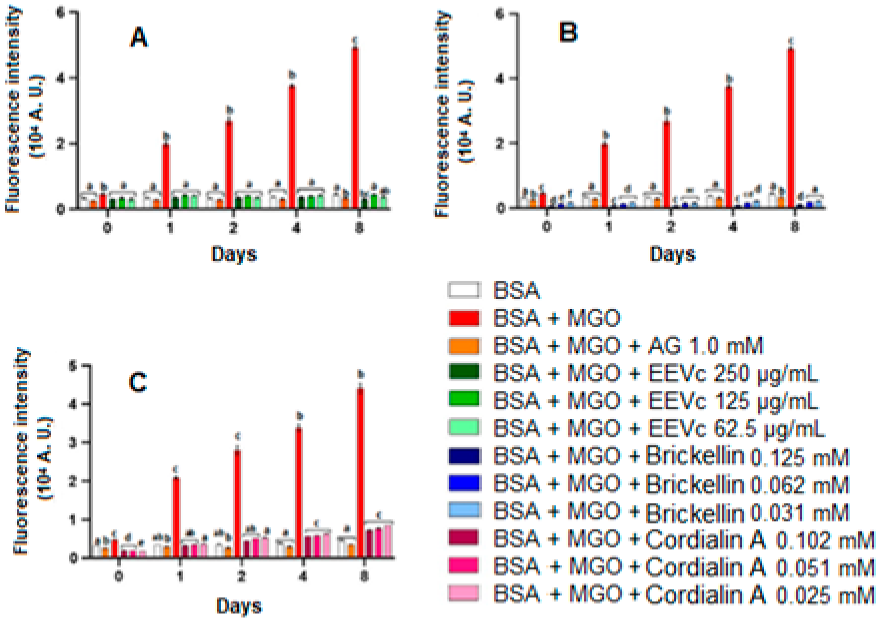

Figure S15: Effects of different concentrations of EEVc (A), brickellin (B) and cordialin A (C) on *N*'-formylkynurenine formation in BSA-only protein glycation model system *in vitro*.

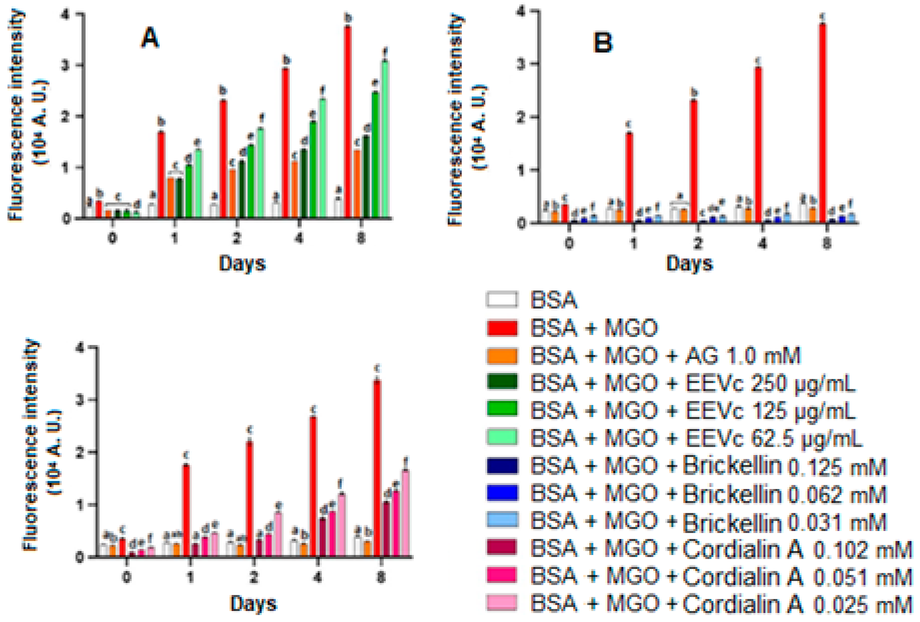

Figure S16: Effects of different concentrations of EEVc (A), brickellin (B) and cordialin A (C) on the formation of Quinurenin in BSA-only protein glycation model system *in vitro*.

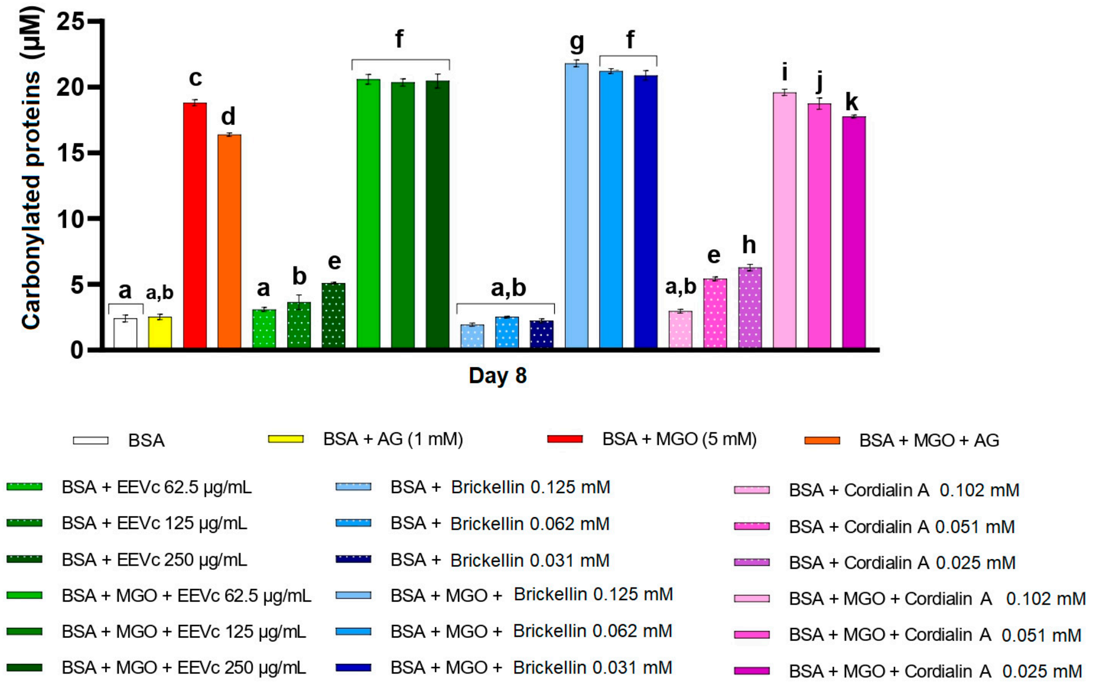

Figure S17: Quantification of carbonylated proteins obtained on day 8 *in vitro* protein glycation model system using BSA and MGO. BSA: bovine serum albumin; MGO: methylglyoxal; AG: aminoguanidine; EEVc: ethanolic extract of *V. curassavica*.

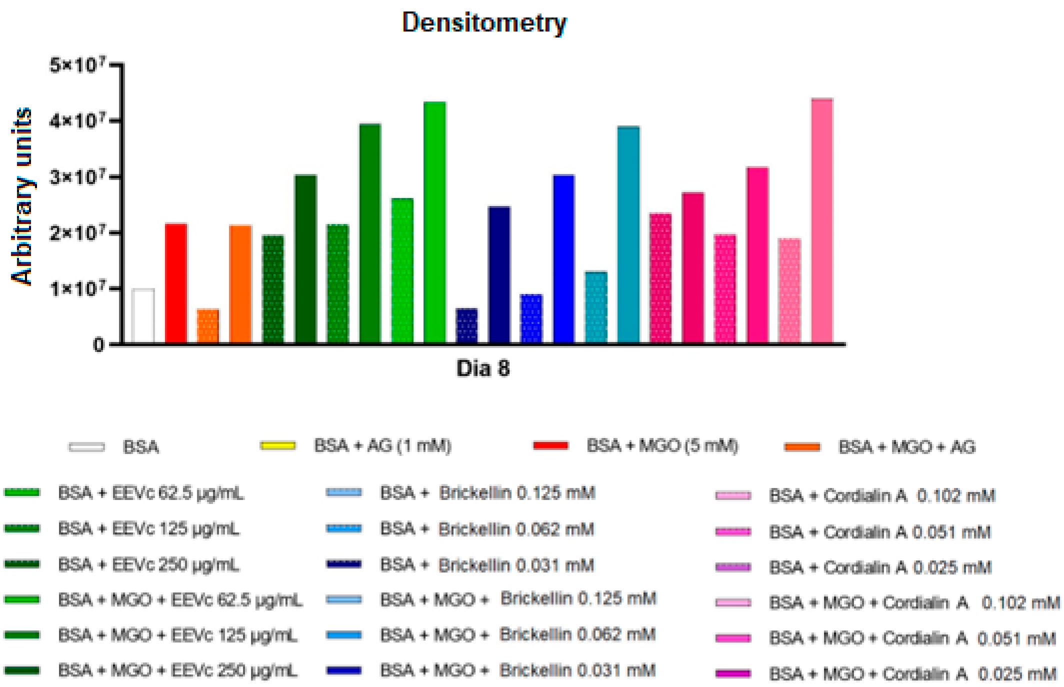

Figure S18: Graphical representation of densitometry calculation generated by ImageJ® 1.53k program regarding crosslinking formation of EEVc, brickellin and cordialin A samples at different concentrations incubated with BSA + MGO on day 8. BSA: bovine serum albumin; MGO: methylglyoxal; AG: aminoguanidine; EEVc: ethanolic extract of *V. curassavica*.
